# Tracing micro and nanoplastics toxicity in human pulmonary fibroblasts through integrated Raman and transcriptomic analyses

**DOI:** 10.1038/s41598-025-22947-7

**Published:** 2025-11-11

**Authors:** Joanna Chwiej, Magdalena Wytrwal, Karolina Papacz, Igor Jasielczuk, Aleksandra Wilk, Artur Gurgul, Tomasz Szmatoła, Ewa Ocłoń

**Affiliations:** 1 Faculty of Physics and Applied Computer Science, AGH University of Krakow, Krakow, Poland; 2 Academic Centre for Materials and Nanotechnology, AGH University of Krakow, Krakow, Poland; 3 Department of Basic Sciences, University of Agriculture in Krakow, Krakow, Poland; 4 Laboratory of Recombinant Proteins Production, University of Agriculture in Krakow, Krakow, Poland; 5 Department of Infectious Disease and Public Health Protection, University of Agriculture in Krakow, Krakow, Poland

**Keywords:** Microplastics, Polystyrene, Toxicity, Human pulmonary fibroblasts, Internalization tracking with raman microscopy, RNA-seq, Transcriptomics, Next-generation sequencing

## Abstract

Inhaled micro- and nanoplastics can reach the distal regions of the lungs, where their elimination is limited due to the lack of efficient clearance mechanisms. Although polystyrene particles have been detected in human lung tissue, the molecular effects of such exposures remain poorly characterized. Understanding the cellular response to microplastics exposure, particularly at the transcriptional and structural levels, is essential for assessing potential health risks. The purpose of this study was to evaluate the impact of primary polystyrene micro- and nanoparticles on human pulmonary fibroblasts, a relevant in vitro model for investigating the molecular mechanisms underlying microplastics-induced pulmonary toxicity. Monodisperse polystyrene particles with diameters of 0.1, 1 and 5 μm were used to evaluate size-dependent internalization and cellular response in human pulmonary fibroblasts. Cells were cultured under standard conditions and exposed to particles in vitro. Internalization and fate of microplastics were tested using Raman microscopy, while transcriptomic alterations were assessed by RNA sequencing to identify early molecular responses associated with particle size. Raman microscopy confirmed the internalization of 0.1 μm polystyrene particles by human pulmonary fibroblasts. Particles sized 1 μm showed a high affinity for the fibroblast cell membrane, however, definitive confirmation or exclusion of their internalization into the cells was not possible due to the sample preparation protocol and measurement conditions used. In contrast, exposure to 5 μm particles resulted in pronounced cytotoxicity across tested concentrations, precluding RNA-seq analysis. Transcriptomic profiling assessed by principal component analysis revealed distinct gene expression patterns in cells following exposure to 0.1 and 1 μm particles. Exposure to 0.1 μm particles led to upregulation of genes involved in mitochondrial function and protein synthesis. In contrast, 1 μm particles caused downregulation of genes associated with oxidative phosphorylation and proteostasis. This study shows that particle size and concentration critically influence the molecular response of human pulmonary fibroblasts to polystyrene micro- and nanoparticles. Raman microscopy proved a valuable tool for detecting particle internalization and assessing size-related biochemical changes, including in the nanoscale range.

## Introduction

 Microplastics (MPs) are solid plastic microparticles that, according to the current legal status of the European Union, meet the established size criteria. Their size ranges from approximately 100 nm up to 5 mm in all dimensions for particles, and from 300 nm to 15 mm in length for plastic fibers. It should be noted that the lower size limits largely reflect current analytical method capabilities and detection limits, rather than strict physical or chemical definitions, meaning that smaller particles may exist but are challenging to detect and characterize with standard techniques^1^. MPs are divided into primary and secondary ones. According to the definition of Song J. et al.^2^, primary MPs are intentionally manufactured in microscopic sizes whilst secondary ones are formed or unintentionally produced from larger plastics fragments after breaking down into smaller particles through the physical, chemical, and/or biological processes.

MPs can enter the human body through various routes. Exposure may occur through the oral route – together with water, drinks and food, through the respiratory one and through the skin^3^. Micro- and nanoplastics (MNPs) have been found in the human body in numerous tissues, including blood^4^, brain^5^, uterus^6^ and placenta^7^. From animal studies it is known that MNPs may cause various undesirable effects from^1^ cellular, such as hepatocyte necrosis, through^2^ tissular, such as inflammation or oxidative damage, and^3^ genetic, e.g. influencing the expression of genes regulating the functioning of the endocrine system, to^4^ toxic, affecting the reproduction, growth or survival of organisms^8^.

The literature on the impact of MNPs on human health usually focuses on the digestive system, which is related to the common occurrence of these pollutants in water and food. However, the oral route of exposure does not seem to be the most dangerous, because organisms have developed methods of removing dangerous and unnecessary substances from the digestive system, as evidenced by, among others, a study confirming the presence of various types of MNPs in all of the tested stool samples^9^. The situation is completely different in the case of plastic microparticles that enter our bodies through the respiratory tract, because apart from the cough reflex or the rhythmic movement of the cilia of the respiratory epithelium, there are no natural mechanisms for self-cleaning of the lungs.

Various polymer particles have been detected in lung samples^10^ as well as in bronchoalveolar lavage fluid^11^. Although existing literature suggests their toxic effects on cell cultures and animal models, the causal link between MNPs and pulmonary or systemic diseases in humans has not been established^12^. Several studies have documented the detrimental effects of MNPs on lung tissue. For instance, Zhang et al. demonstrated that inhalation of polystyrene (PS) MNPs resulted in pulmonary fibrosis^13^. Similarly, it has been proven that inhalation of PS MNPs may induce emphysema^14^. Limited available epidemiological studies also suggest that disorders like lung nodules and asthma may be caused by MNPs^15^.

Human pulmonary fibroblasts (HPFs) play a crucial role in maintaining the structural integrity of lung tissue and the response to injury. HPFs are involved in extracellular matrix production, tissue remodeling, and fibrosis process, and therefore are a highly appropriate model for studying the potential pathological effects of inhaled MNPs. Recent studies have shown that polyvinyl chloride (PVC) and polystyrene (PS) microparticles, can induce oxidative stress and mitochondrial dysfunction in lung epithelial cells, triggering in them the senescence and inflammatory signaling pathways^16,17^. These processes are central to the development of fibrosis, in which fibroblasts are key effector cells. Additionally, MNPs have been shown to significantly alter the morphology and proliferation of lung cells, without necessarily inducing cell death, suggesting long-term structural consequences^13^. Summarizing, HPFs are a human-relevant in vitro model for exploring molecular mechanisms of MNP-induced pulmonary toxicity and therefore they were chosen for this study.

Detection, identification and quantification of MNPs in laboratory and environmental samples is crucial to understand their precise behavior and interactions, and potential toxic effects on diverse biological systems^18^. The methods used for MNPs characterization include visual analysis, particle analysis by laser diffraction, dynamic light scattering, scanning electron microscopy, Fourier-transform infrared (FTIR) and Raman spectroscopy/microscopy, thermal analysis, mass spectrometry and flow cytometry^19,20^. Each of these methods allows us to get specific information about MNPs and has both advantages and disadvantages. The most frequently used is visual method which is useful for rough classification and counting of the larger MNPs according to the color, shape and size^21,22^. Although, this method is easy, cheap and safe, it does not provide information on the chemical composition of MNPs^23^, it is time-consuming, and its results may be easily affected by impurities in samples, the color, shape and structure of MNPs as well as individual subjective judgment of the laboratory staff^23,24^.

In this study, Raman microscopy was employed to monitor the internalization and fate of polymer particles of micrometer and submicrometer sizes in cells. Compared to the complementary FTIR microspectroscopy, Raman microscopy offers significantly higher spatial resolution, enabling the detection and localization of particles in the submicron range, which is beyond the capabilities of FTIR microspectroscopy. Raman microscopy allows the investigation of MNPs without the need for special markers, in a biological environment and under conditions close to the natural state, which is often crucial for studying particle-cell interactions^19,22^. This technique is also less affected by spectral interferences and can therefore effectively register signals from particles located inside cells, even when they partially overlap with the biological background. These features make Raman microscopy an optimal tool for studying the internalization and tracking the fate of polymer particles in cells at the micro- and nanoscale^25–27^.

The purpose of the present study was to evaluate the impact of primary PS micro- and nanoparticles (PS-MNPs) on HPFs being a relevant in vitro model for exploring molecular mechanisms of MNPs-induced pulmonary toxicity. The effect of PS-MNPs with three different diameters (0.1, 1 and 5 μm) was examined in this paper. To distinguish between micro- and nanoplastics, we applied a classification in which microplastics (MPs) are defined as polymer particles ranging from 1 μm to 5 mm, and nanoplastics (NPs) as particles smaller than 1 μm. This allowed us to verify if MNPs influence on cell viability, and cytotoxicity and apoptosis levels are particle size-dependent. The toxicity tests were performed for various doses (0.1, 0.05, 0.01, 0.005, 0.001, and 0.0005 g/L) and exposure times (6, 24 and 48 h). Their results were the base of selection of the experimental conditions for further transcriptomic studies whose purpose was to identify cellular pathways and signaling cascades involved in the molecular mechanisms underlying MNPs toxicity to HPFs.

## Materials and methods

**Fig. 1:**
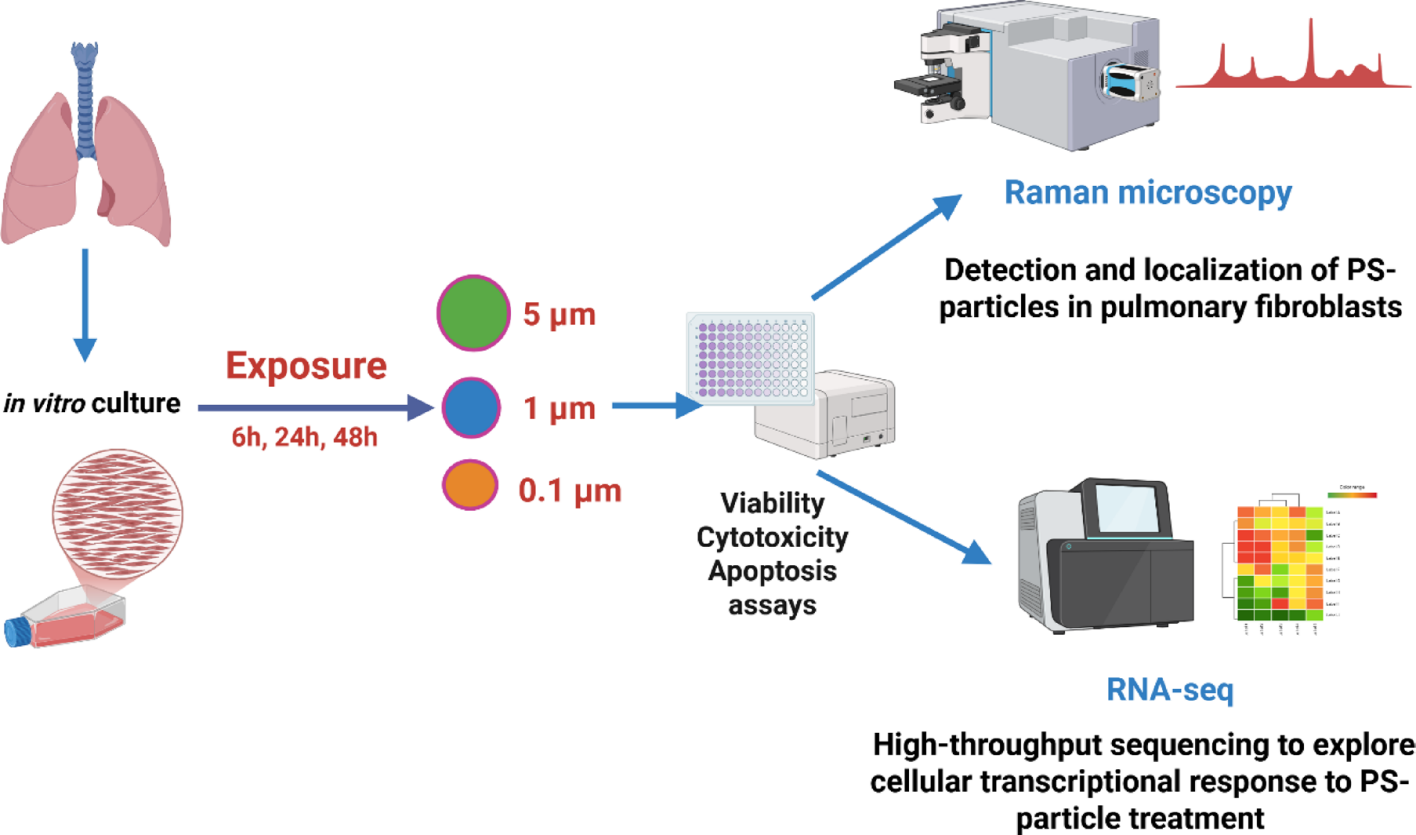
Schematic overview of the experimental design (Created with BioRender.com).

A schematic overview of the study design, including exposure conditions and analytical approaches, is presented in Fig. 1.

### Microplastics

To examine the influence of MNPs on HPFs, we used particle size standards based on monodispersed polystyrene (PS, cat. no.: 43302, 89904, and 79633; MERCK). According to the manufacturer, particle diameters were 0.101 ± 0.007 μm (coefficient of variation, CV = 6.7%), 0.95 ± 0.03 μm (CV = 2.7%), and 5.07 ± 0.09 μm (CV = 1.7%), respectively. The MNPs were used without chemical surface modifications but were sterilized prior to experiments using UV radiation to eliminate potential microbiological contaminants. For sterilization, 0.1 mg/mL suspensions of PS particles in deionized water were placed in quartz cuvettes on a magnetic stirrer and irradiated with a UV lamp (HNS™ 30 W G13, λ = 254 nm, OSRAM) for 15 min at a distance of 75 cm. All working dilutions, including those used for cytotoxicity and transcriptomic analyses, were prepared from this stock suspension.

Dynamic light scattering (DLS) measurements (Malvern Zetasizer Nano ZS, A. P. Instruments) of average diameter, polydispersity index, and ζ-potential, taken before and after UV irradiation, indicated no effect of the sterilization procedure on these parameters. Furthermore, Raman spectroscopy revealed no significant changes in the spectral characteristics of the particles, confirming that the structural properties of the MNPs were also preserved after this process.

### Cell culture and treatments

HPFs were obtained from PromoCell GmbH (cat. no.: C-12360; Germany) and cultured in Dulbecco’s Modified Eagle Medium (DMEM, High Glucose; Thermo Fisher Scientific, USA), supplemented with 10% fetal bovine serum (FBS; Thermo Fisher Scientific, USA) and an antibiotic-antimycotic solution containing penicillin (10,000 U/mL), streptomycin (10,000 µg/mL), and amphotericin B (25 µg/mL; Thermo Fisher Scientific, USA). Cells were maintained at 37 °C in an atmosphere containing 5% CO₂.

To assess the cellular effects of PS micro- and nanoparticles, HPFs were exposed to spherical PS particles of three different diameters. For cell viability and apoptosis/toxicity assays (ApoTox-Glo™ Triplex Assay; Promega)^28^, cells were seeded into 96-well plates at a density of 15,000 cells per well and exposed for 6, 24, and 48 h to MNPs. Six concentrations of PS particles were tested for each particle size: 0.1 g/L (A), 0.05 g/L (B), 0.01 g/L (C), 0.005 g/L (D), 0.001 g/L (E), and 0.0005 g/L (F). The concentration range used in this study is consistent with previously published in vitro models employing PS micro- and nanoparticles. For 100 nm particles, comparable exposure levels (0.005–0.1 g/L) have been reported to induce uptake, oxidative stress, and genotoxicity in human cell lines^29,30^. For 1–5 μm particles, similar concentrations (0.001–0.01 g/L) were shown to affect viability and generate ROS in fibroblasts and neuronal cells^31,32^. All treatments, including untreated controls, were conducted in triplicate to estimate the technical variability. Results for viability are expressed as a percentage of the control signal (control = 100%), whereas cytotoxicity and apoptosis are expressed as a percentage of the maximum assay signal (control = 0%).

For transcriptomic analysis, cells were seeded in 12-well plates at a density of 150,000 cells per well and incubated for 24 h in selected exposure conditions. Based on the results of the ApoTox-Glo™ assays, two particle sizes − 100 nm and 1 μm - were selected for transcriptomic profiling, as they showed consistent viability levels at 24 h and induced detectable cytotoxic and apoptotic responses without compromising RNA quality. The 5 μm particles were excluded from transcriptome studies due to their pronounced cytotoxicity across most tested concentrations, which resulted in excessive cell death and precluded reliable analysis of gene expression profiles. For the 100 nm particles, three concentrations were selected: 0.1 g/L (MA100), 0.01 g/L (MB100), and 0.001 g/L (MC100). For the 1 μm particles, four concentrations were included: 0.1 g/L (MA1), 0.01 g/L (MB1), 0.001 g/L (MC1) and 0.05 g/L (MD1). This selection enabled dose-dependent analysis of particle-induced transcriptional changes under viable conditions.

### Raman measurements

For Raman measurements, the cells were first cultured in standard plastic petri dishes, counted using a Bürker chamber and then seeded onto sterilized CaF_2_ windows (diameter of 25 mm, thickness of 2 mm; Crystran LDT, UK) with the density value of 75,000 cells per well. Before seeding the cells, the windows were cleaned for 5 min in a plasma cleaner (PDC-32G-2) and then sterilized under UV for 30 min. After 48 h, when the cells had adhered to the surface of the plates, the stimulation protocol with PS particles was initiated. UV sterilized particles with the diameter of 0.1, 1 and 5 μm were added to cells in the concentration of 0.1 mg/mL. After 48 h exposure time, the medium was removed, the cells were washed twice with PBS solution and fixed with 4% paraformaldehyde. After fixation, they were washed three times with deionized water and dried under the laminar chamber.

Raman measurements were performed in the Atomic and Molecular Biospectroscopy Laboratory (Faculty of Physics and Applied Computer Science, AGH University of Krakow) using a WITec Alpha300 R confocal Raman imaging microscope equipped with a 600 grooves/mm diffraction grating. The excitation source was a 532 nm laser set to 7 mW, with an integration time of 0.5 s and 3 spectral accumulations per measurement point. The cells were subjected to raster scanning with the laser beam using the step size which depended on the MNPs utilized for stimulation. In the case of 5 μm PS particles, the step was equal to 1 μm whilst for smaller 0.5 μm. For each condition, 10 HPFs were measured and compared with the appropriate control cells.

### Transcriptome analysis

Upon completion of the treatment duration, cells were subjected to trypsinization, transferred into centrifuge tubes, and centrifuged at 500 relative centrifugal force (RCF) for 5 minutes to form cell pellets. The supernatant was then discarded, and the cell pellets were snap-frozen at − 80°C for storage until RNA extraction. Total RNA was isolated using the standard TRI Reagent™ Solution protocol (ThermoFisher Scientific, USA). The quality of the isolated RNA was assessed with the TapeStation 4150 System (Agilent, USA), and RNA quantification was performed using the Qubit RNA Broad-Range (BR) assay (ThermoFisher Scientific, USA). Library preparation was conducted using 50 ng of total RNA with the QuantSeq 3’ mRNA-Seq Library Prep Kit FWD (Lexogen, Austria), which generates a single fragment per transcript at the 3’ end, thereby reducing the number of required raw reads per sample to approximately 3 million. The quality of the indexed libraries was verified with the TapeStation 4150 System (Agilent, USA) and quantified using the Qubit double-stranded DNA (dsDNA) BR kit (ThermoFisher Scientific, USA). Subsequently, the library pools were sequenced commercially at the OMRF Clinical Genomics Center (CGC) using a paired-end 150 bp run on the Novaseq6000 System (Illumina, USA) to obtain at least 6–7 million reads per sample. The resulting raw sequences from the first sequencing read and read counts were deposited in the Gene Expression Omnibus (GEO) and Sequence Read Archive (SRA) databases of the National Center for Biotechnology Information (NCBI) under the accession number GSE296007.

### Bioinformatic analysis

The obtained raw sequencing reads were quality-checked using FastQC software (v0.11.9). Initially, the second sequencing read was discarded, as only the first read is required for gene expression analysis with the used 3’mRNA-Seq approach. Filtering and trimming of the first read were performed with Flexbar software (v3.5.0)^33^, where low-quality read ends, adapter sequences, and excessively short reads post-trimming were eliminated. The cleaned high-quality reads were then aligned to the human GRCh38 genome assembly using the STAR aligner software (v2.7.5c)^34^. Read counts were quantified using the MM109 (Ensembl) annotation and Htseq-count software (v1.99.2)^35^. Normalization of read counts and differential expression (DE) analysis were conducted using DESeq2 software implemented in iDEP2.0 server^36^, with a false discovery rate (FDR) threshold of < 0.05 to identify significant DE genes. Functional enrichment analysis of the DE genes was performed for specific gene ontology biological processes (GO BP) and Kyoto Encyclopedia of Genes and Genomes (KEGG) pathways^37^ using the iDEP2.0 server^38^.

### Validation of RNA-Seq gene expression by droplet digital PCR

To validate RNA-seq expression data for *SOX4* (Hs04987498_s1), *SOX9* (Hs00165814_m1), and *FOXC1* (Hs00559473_s1), droplet digital PCR (ddPCR) was performed using the QIAcuity digital PCR system (Qiagen, Germany). Duplex ddPCR reactions were assembled using FAM-labeled TaqMan™ Gene Expression Assays (Thermo Fisher Scientific, USA) for each target gene and normalized to the endogenous control *HPRT1*, detected with a VIC™/TAMRA™-labeled probe (Human HPRT1 Endogenous Control, Primer Limited; Thermo Fisher Scientific, USA). Each 12 µL reaction contained 2 µL of cDNA template, QIAGEN Probe PCR Kit reagents (Qiagen, Germany), and primers/probes according to the manufacturer’s instructions. Reactions were dispensed into QIAcuity Nanoplate 8.5k 24-well plates (Qiagen, Germany) designed for high multiplex digital PCR. Thermal cycling was performed under the following conditions: enzyme activation at 95 °C for 2 min, followed by 40 cycles of 95 °C for 15 s (denaturation) and 60 °C for 30 s (annealing/extension). Imaging was conducted with an exposure time of 500 ms and a gain of 6 for both the green (FAM) and yellow (VIC) channels. The HM-Ref reference channel was enabled during imaging. Absolute quantification of target copy numbers was performed using QIAcuity Software Suite version 3.1.0.0 (Qiagen, Germany), based on Poisson distribution statistics.

## Results

### Internalization of PS MNPs into HPFs

**Fig. 2:**
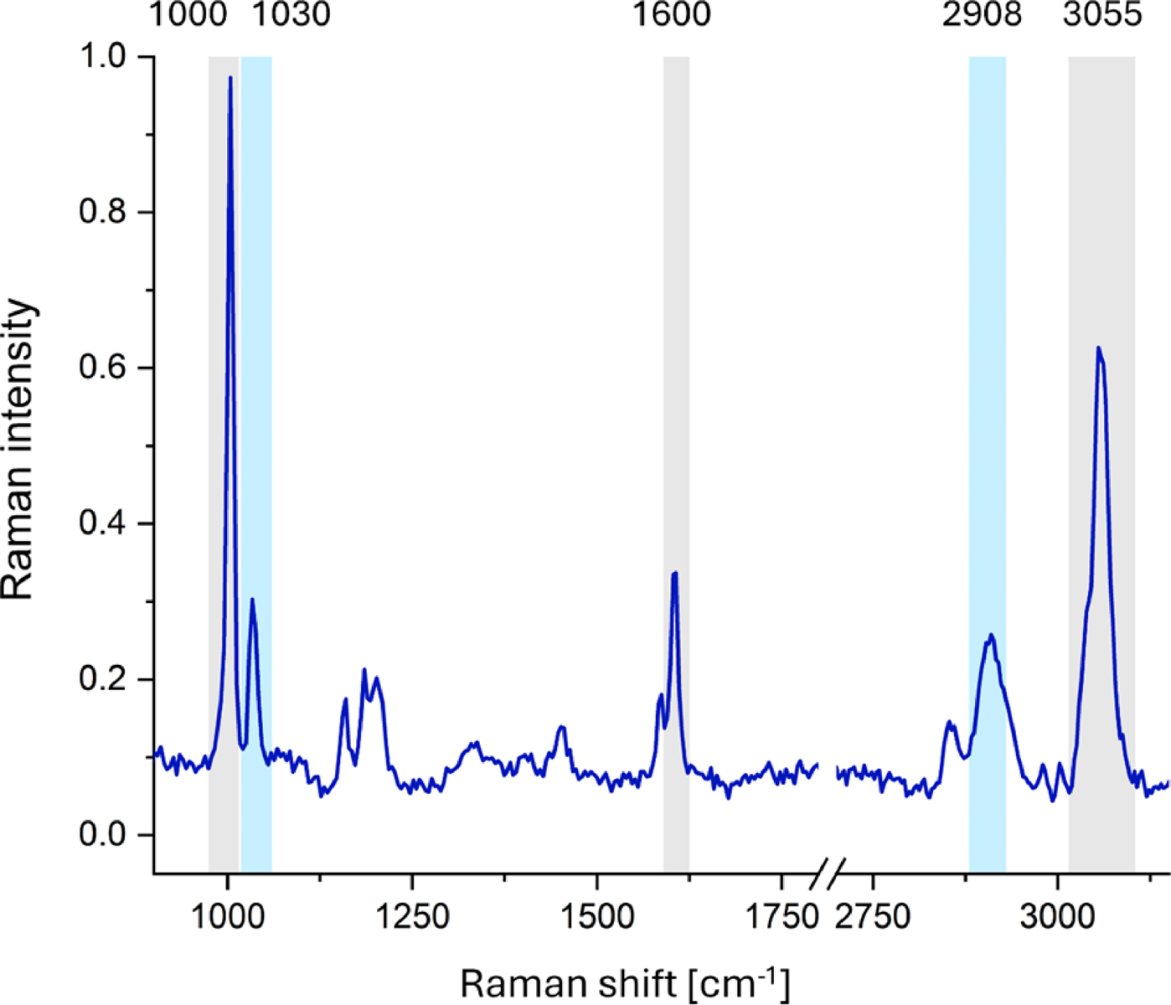
The Raman spectrum of PS MNPs used in the study.

**Fig. 3:**
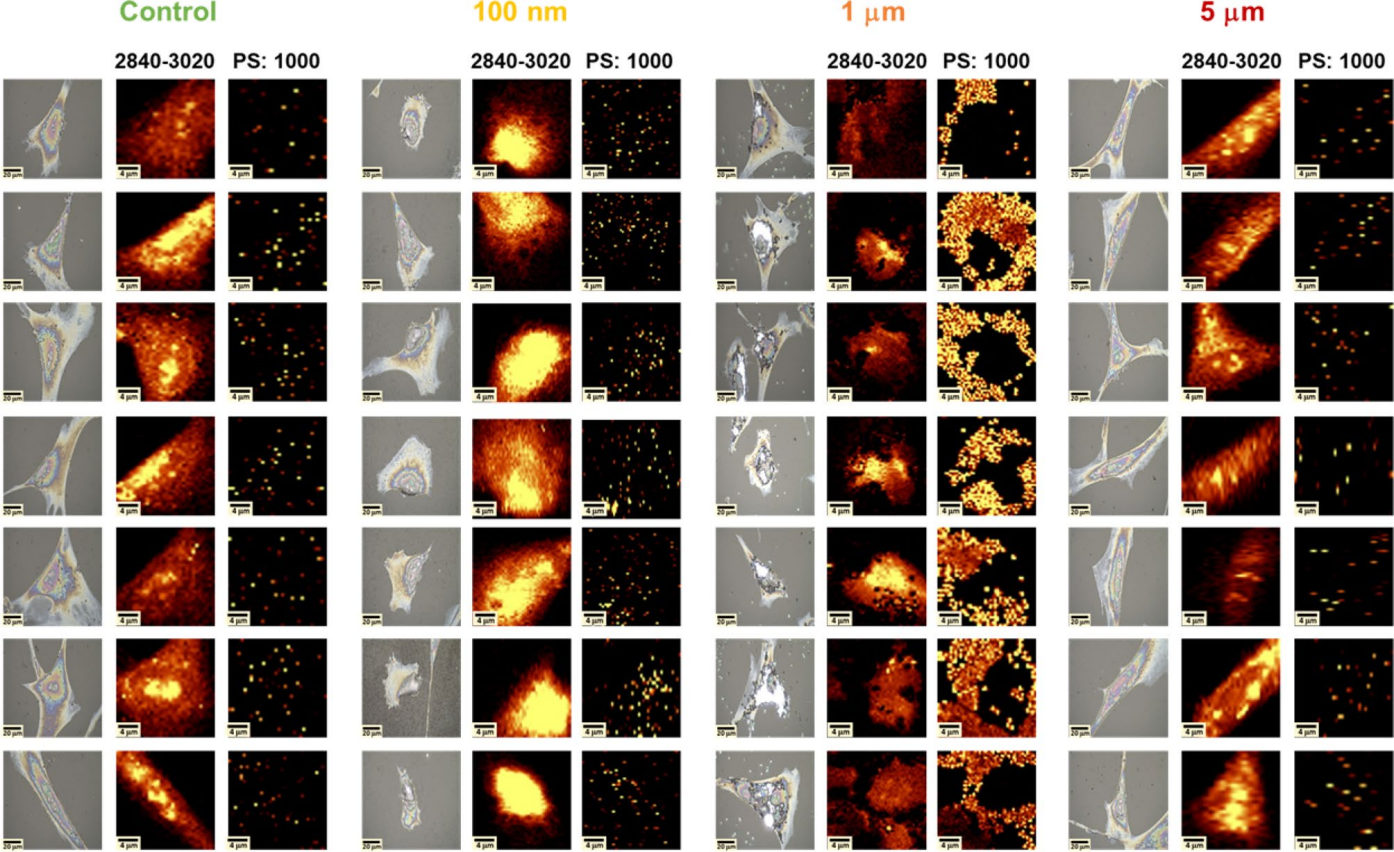
Comparison of microscopic images with 2D Raman chemical maps showing the distribution of signals specific to cellular organic matter (2840–3020 cm⁻¹) and polystyrene (PS: 1000 cm⁻¹) in representative HPFs. From left to right: control cells and cells exposed to 0.1, 1, and 5 μm PS particles. Microscopic images and chemical maps were acquired using a WITec Alpha300R Raman microscope equipped with a 100× objective. Scale bars: 20 μm for microscopic images and 4 μm for Raman maps.

To verify whether exposure to MNPs can lead to their internalization into HPFs, Raman microscopy was employed. As shown in Fig. 2, the most prominent Raman bands in the PS spectrum appear at the wavenumbers of around 1000, 1030, 1600, 2908, and 3055 cm⁻¹. The origin of these bands is described in Table 1. In Fig. 3, the results of the topographic analysis of the band at 1000 cm⁻¹ - characteristic of PS - are compared with the chemical maps illustrating the signal intensity distribution within the spectral region of 2840–3020 cm^− 1^. The 1000 cm^− 1^ band corresponds to ring-mode vibrations typical of monosubstituted aromatic compounds^25,39,40^, whereas the broader region at higher wavenumbers primarily reflects organic components typical of cellular material. As shown in Fig. 3, for HPFs exposed to 5 μm particles, the distribution of the PS-specific band shows no noticeable differences compared to control cells. In contrast, the presence of MNPs in the scanned area can be unequivocally confirmed in the chemical maps of HPFs exposed to 1 μm particles. This effect is manifested by a pronounced increase in the Raman signal at around 1000 cm^− 1^ and a local decrease in the signal originating from cellular organic matter (2840–3020 cm^− 1^). Although, mentioned chemical maps reveal high affinity of 1 μm particles to the membranes of HPFs, they do not allow for clear confirmation or exclusion of the internalization of these particles into the cells. Attempts to perform depth scans at selected points within the cells also did not provide a definitive answer regarding particle penetration. This limitation is attributed to sample preparation, specifically the drying of cells prior to measurement, which reduces the cell thickness so much that the polymer signal is detected throughout nearly the entire cell depth. This challenge could potentially be addressed in the future by performing measurements on cells in an aqueous environment using an immersion objective.

**Fig. 4:**
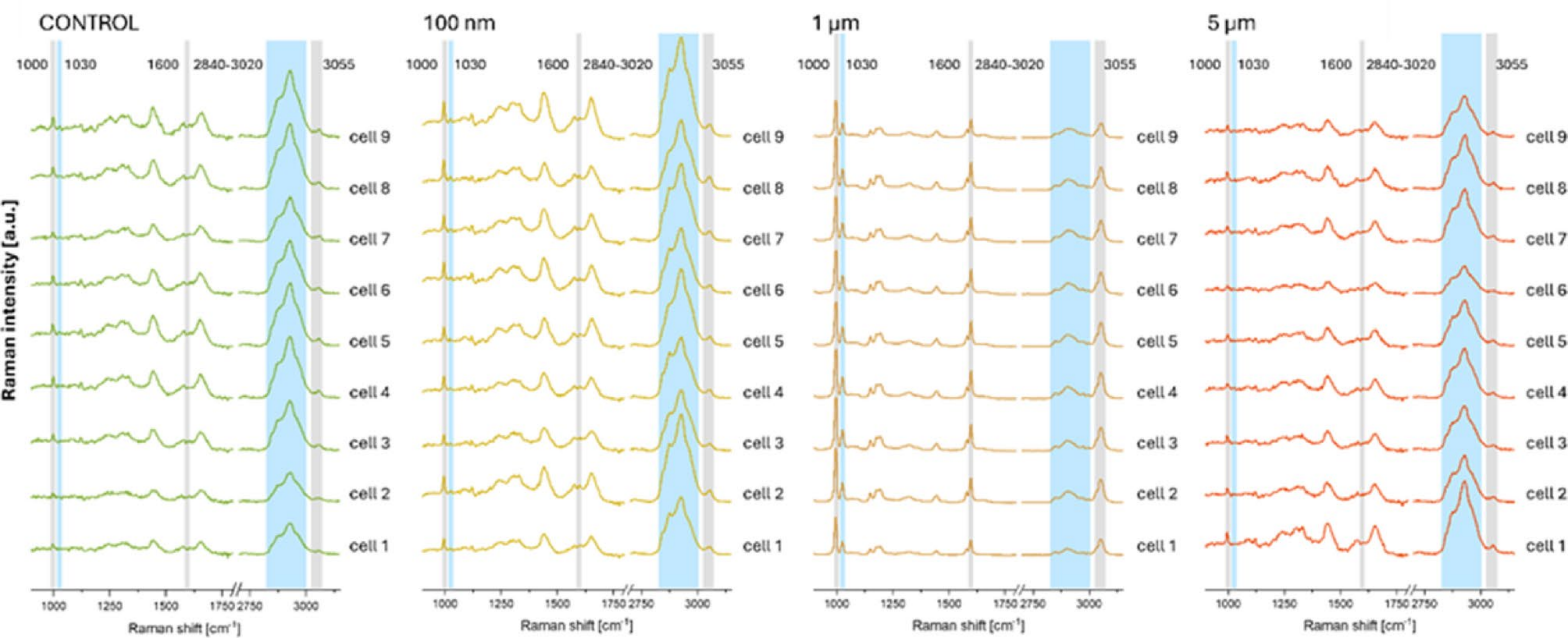
The comparison of average Raman spectra recorded for control HPFs and those exposed to 100 nm, 1 μm and 5 μm PS particles. The spectra were baseline corrected and multiplied by factor 2 in the fingerprint region.

The chemical maps obtained for 100 nm PS particles are characterized by a larger number of points presenting increased Raman signal at the wavenumber of 1000 cm^− 1^ and enhanced accumulation of organic matter within HPFs. Both effects may result from the internalization of NPs into cells as an intensive Raman signal from PS occurs not only at the wavenumber of 1000 cm^− 1^ but also around 2930 cm^− 1^. To verify this, the average Raman spectra were calculated for cells exposed to PS particles of various sizes and control HPFs. The obtained results are compared in Fig. 4. As it was expected, the average Raman spectra of cells treated with 1 μm MPs strongly differ from those of control HPFs. Such differences were not found for HPFs subjected to the action of 5 μm particles. For cells exposed to the smallest particles, the spectral data indicate a possible internalization of PS. The average spectra of cells treated with 100 nm PS particles present increased intensity of the signal at the wavenumbers of 1000 and 3055 cm^− 1^ as well as at the region specific for organic matter. To confirm that the found anomalies result from the presence of PS in HPFs, the cluster analysis was performed based on the Raman spectra recorded for these cells. The squared Euclidean distance was used as a measure of similarity between the spectra, and Ward’s method was utilized for their unsupervised hierarchical classification.

**Fig. 5:**
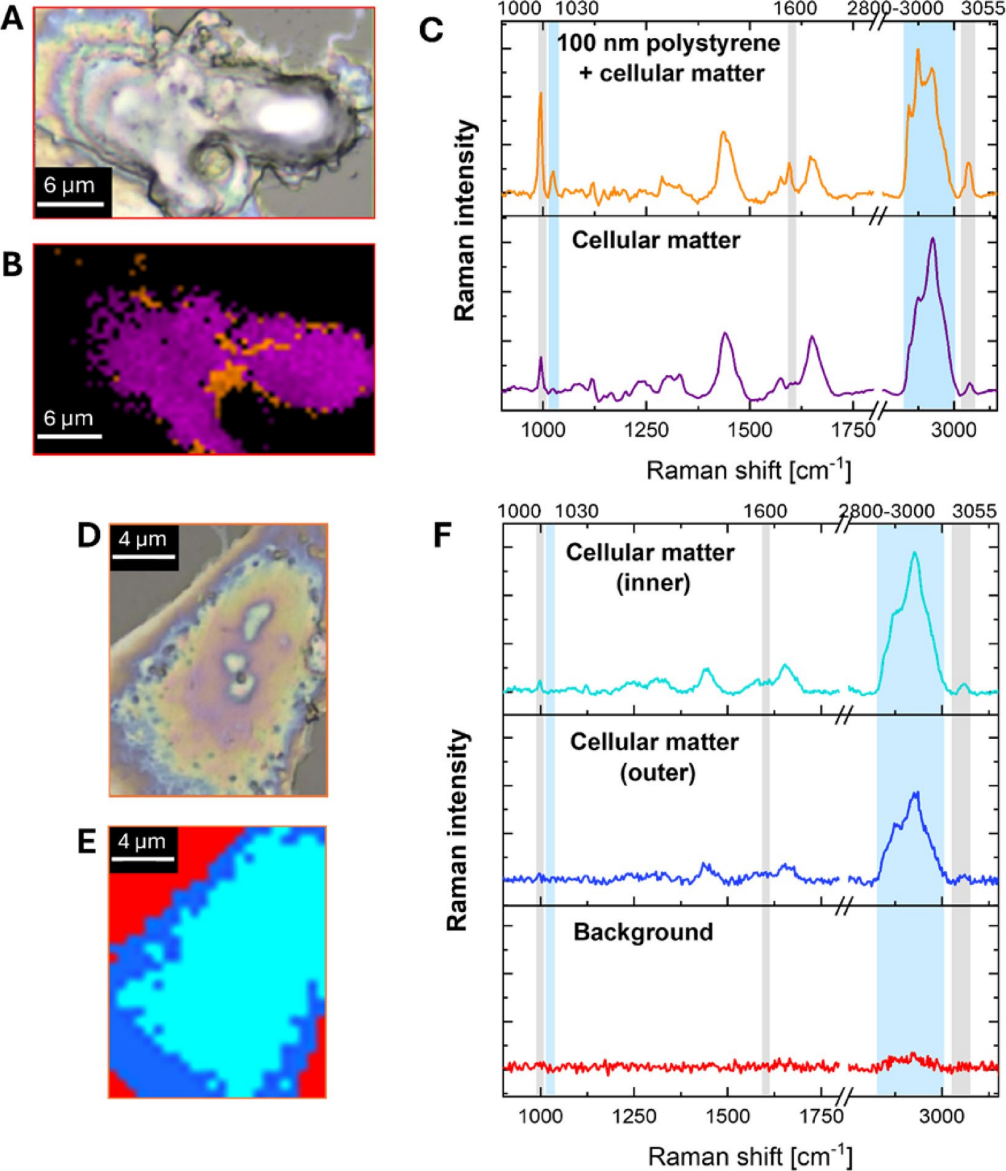
The results of cluster mapping (squared Euclidean distance, Ward’s method) performed for selected HPF subjected to the action of 100 nm PS particles. (**A**) microscopic picture of the cell, (**B**) cluster map, and (**C**) average Raman spectra of obtained clusters. Analogous data for the control cell are presented in (**D**) microscopic image, (**E**) cluster map, and (**F**) average Raman spectra of the clusters.

The cluster map for a representative HPF cell exposed to 0.1 μm PS particles is shown in Fig. 5B. The corresponding microscopic image and the average Raman spectra of the two identified clusters are presented in Fig. 5A and C, respectively. Analogous data for the control cell are shown in Figs. 5D-F. The mean spectrum of cluster 2 in the exposed cell (Fig. 5C) exhibits markedly increased signal intensities at wavenumbers characteristic of PS. The pronounced Raman bands at 1000, 1030, 1600, 2908, and 3055 cm^− 1^ indicate the presence of larger assemblies of PS particles within the cell. The spectrum of cluster 1 in the exposed cell also subtly differs from those obtained for control cell (Fig. 5F) and likewise points to the internalization of PS.

In summary, the Raman study results demonstrated that PS particles with a size of 5 μm did not undergo internalization into HPFs. In contrast, particles with a diameter of 1 μm exhibited high affinity for cell membranes; however, their internalization could not be unequivocally confirmed due to limitations associated with the applied cell preparation method. For the smallest particles (100 nm), both chemical mapping and spectral as well as cluster analyses revealed the presence of characteristic PS bands inside the cells, which unequivocally confirmed the penetration of these particles into HPFs.

**Table 1:** The assignment of the bands measured in Raman spectra of MNPs to the vibrations of bonds or functional groups of PS.

Band [cm^–1^]	Assignment	Structural origin	References
1000	(1) C-C stretching (2) in-plane ring breathing (characteristic of a single-substituent aromatic compound)	(1) bond between C atoms in the carbon chain (2) phenyl group	^25,39,41^
1030	C-H in-plane ring breathing	phenyl group	^25,41^
1600	(1) C-C symmetric ring stretching (2) C-C-H in-plane ring bending	phenyl group	^25,41^
2908	CH_2_ stretching	methylene group of carbon chain	^25,42^
3055	CH_2_ aromatic stretching	phenyl group	^40,25^

### Effect of microplastics on the viability, cytotoxicity and apoptosis level of HPFs

**Fig. 6:**
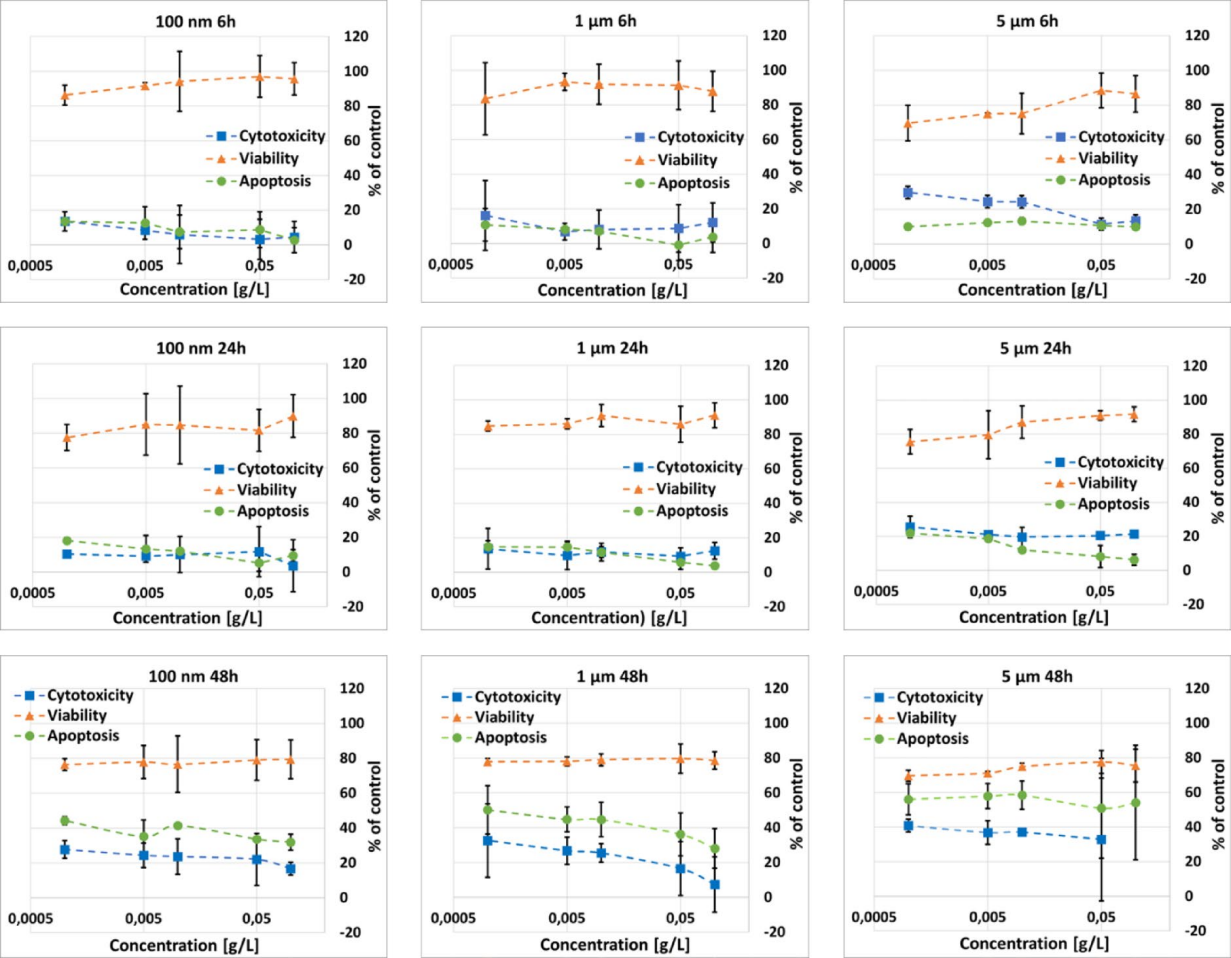
Dependencies of the viability, cytotoxicity and apoptosis levels on MNPs concentration for examined particle sizes (100 nm, 1 and 5 μm) and exposure times (6, 24 and 48 h).

The results of the ApoTox-Glo™ test are shown in Fig. 6. As one can see, all examined MNPs influenced the viability, cytotoxicity and apoptosis level of HPFs cells as expressed relative to untreated controls. The viability of cells exposed for 6 h to 100 nm PS particles was approximately 90–95% of the control. 24-hour exposure to these particles caused the decrease of viability to around 80%. This value remained unchanged after the next 24-hour long incubation period. Cytotoxicity and apoptosis levels were approximately 10% of the control values at both 6 and 24 h of observation. At 48 h, cytotoxicity increased to 20–25% and apoptosis to 35–40% of the control.

The viability of HPFs exposed for 6 and 24 h to 1 μm PS particles was approximately 90% of the control. A further decrease, to approximately 80%, was observed after the next 24 h of incubation. Cytotoxicity and apoptosis remained at around 10% of control after 6 and 24 h of exposure. Their levels increased to 20–25% and 25–45% (respectively) of the control in the last examined time point.

Following exposure to 5 μm PS particles for 6 h, HPFs viability declined to 75–85% of the control level. This value was maintained at 24 h of exposure and then slight reduction of viability, to around 75–80%, was noticed after the next 24 h of the observation. The cytotoxicity and apoptosis levels in HPFs exposed to the largest PS particles for 6 and 24 h were in the range of 10–25% of control. At 48 h of incubation, the cytotoxicity increased to 35–40%, whilst apoptosis to 55–60% of the control signal.

Overall, a decrease in cell viability relative to the control was observed at all the tested experimental conditions, with an average reduction of this parameter of approximately 15%. The highest decrease in viability and increase in cytotoxicity were recorded in the case of 48 h of exposure. Among all particle sizes tested, the highest apoptotic response was observed in HPFs exposed for 48 h to 5 μm PS particles.

### Sequencing statistics and general genes expression profile differentiation

For the 3’mRNA-Seq experiment, more than 620 M sequencing reads were generated for 24 samples — around 26 M per sample. Of the raw reads, on average 73% were uniquely mapped against the newest available reference genome sequence. For individual samples, 58.1% to 64.3% of the mapped reads were located within annotation features (genes) (Supplementary File 1). Gene-related read counts were subsequently employed for differential expression analysis.

**Fig. 7:**
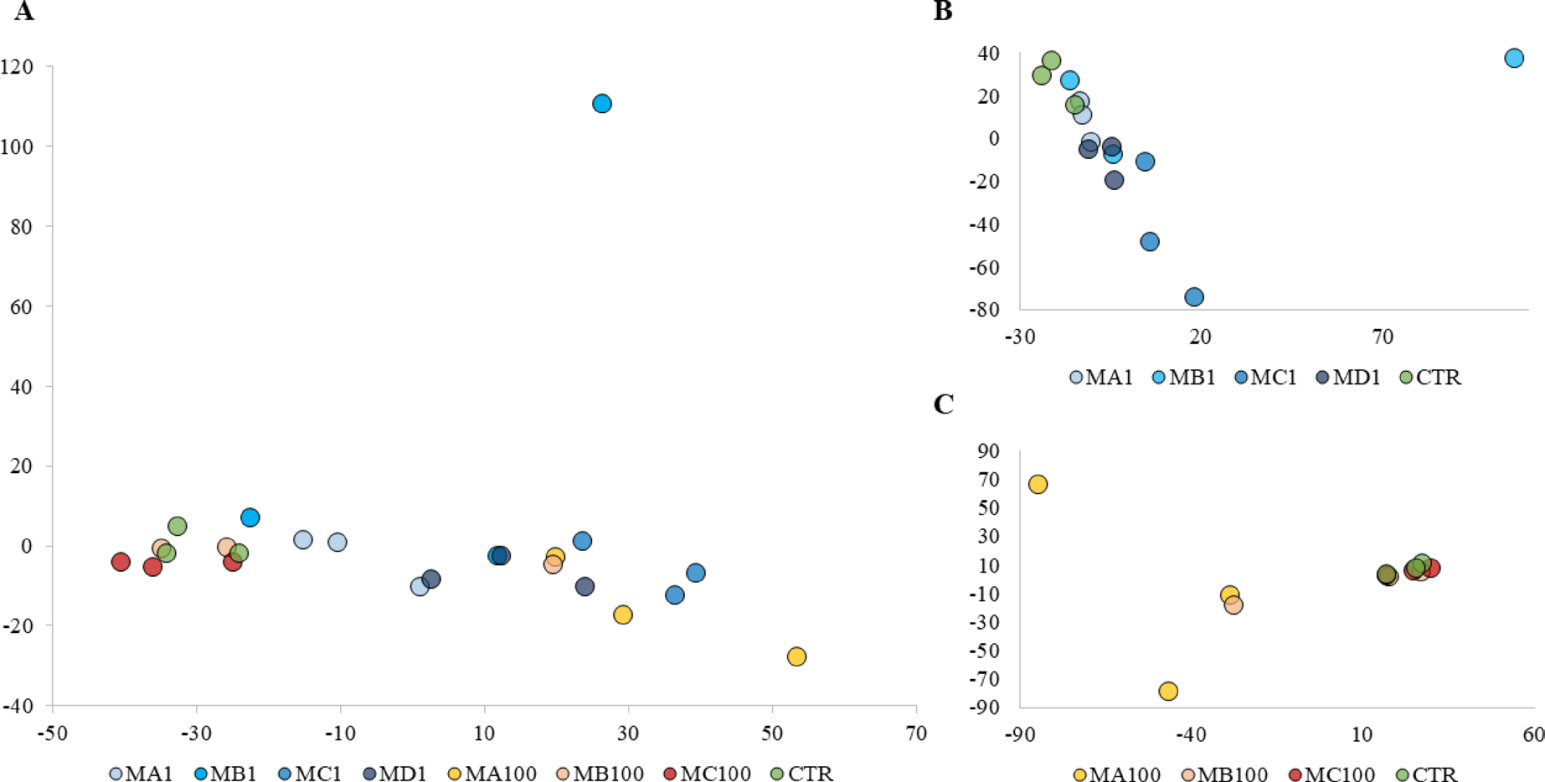
Expression profile differentiation using principal components analysis (PCA) method among HPF cells treated with different concentrations of 1 μm and 100 nm PS particles. (**A**) – PCA among HPFs treated with different concentrations of 1 μm and 100 nm particles; (**B** and **C**) – PCA among HPFs treated with different concentrations of 1 μm and 100 nm particles, respectively. Group labels indicate particle size and concentration: for 100 nm particles – MA100 (0.1 g/L), MB100 (0.01 g/L), MC100 (0.001 g/L); for 1 μm particles – MA1 (0.1 g/L), MB1 (0.01 g/L), MC1 (0.001 g/L), and MD1 (0.05 g/L).

Global gene expression profile evaluation with principal components analysis (PCA) showed that PS particles of 100 nm and 1 μm in different concentrations caused various changes in the transcriptomes of HPFs. The greatest changes compared to the control group were observed in the MA100, MC1, and MD1 groups clustering relatively close to each other. The MC100 group and the MB100 group showed the smallest changes compared to the control group, with one sample separating from the others in this group. The most heterogeneous was the MB1 group, which was characterized by a large differentiation of expression profiles compared to the other groups (Fig. 7).

**Fig. 8:**
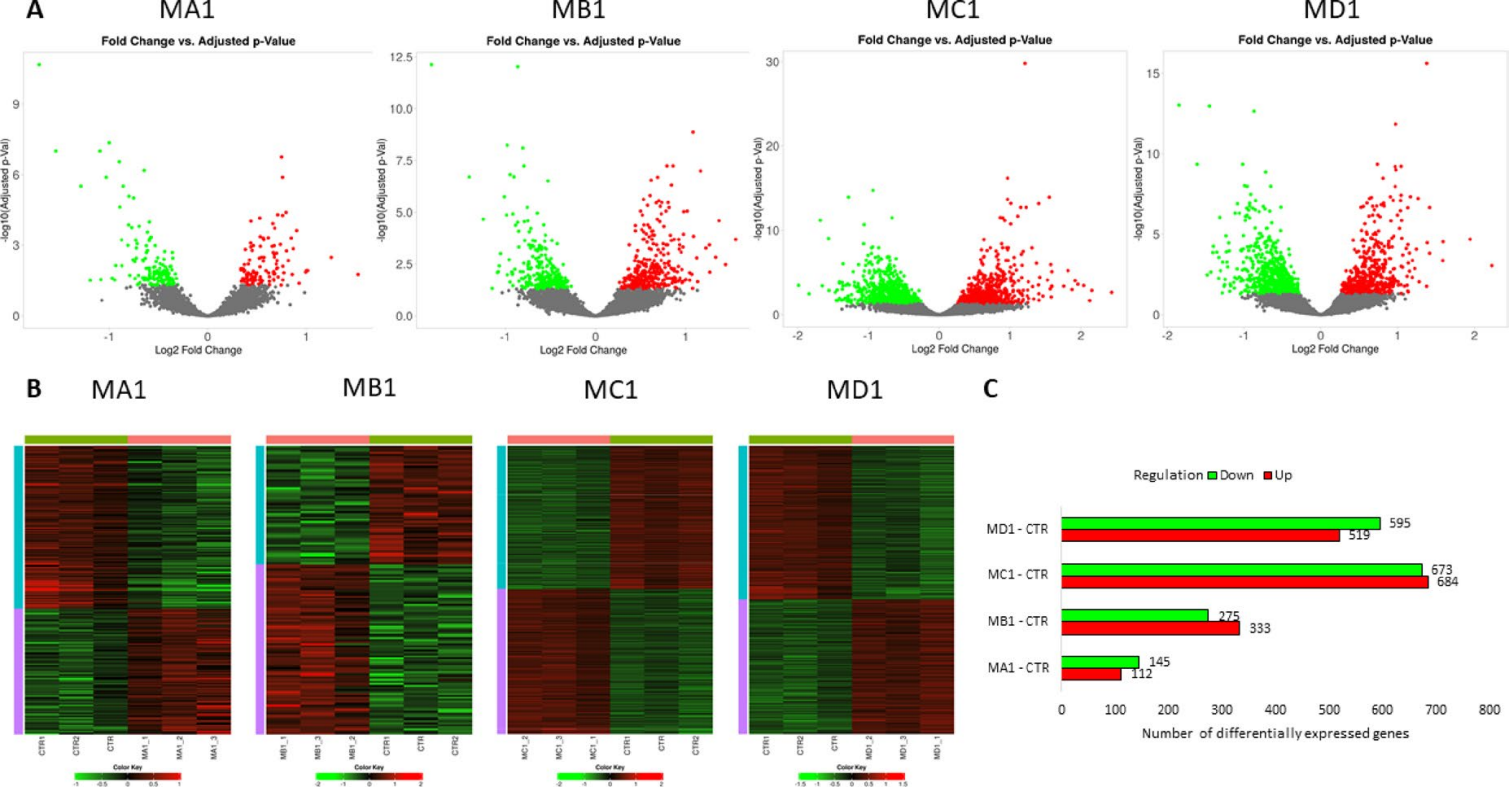
Results of differential expression analysis among HPF cells treated with different concentrations of 1 μm PS particles. (**A**) – Volcano plot for all analyzed genes (significantly altered genes (FDR < 0.05) are marked in red/green); (**B**) – Heatmaps for differentially expressed genes; (**C**) – Number of differentially expressed genes and their regulation.

Among the groups treated with 1 μm PS particles, the MC1 group had the highest number of genes with changed expression compared to the CTR (*n* = 1357), and the MA1 group had the smallest number (*n* = 257) (Supplementary File 2). In the MA1 and MD1 groups, the majority of differentially expressed genes (DEGs) were upregulated (56.4% and 53.4%, respectively), whereas in the MB1 and MC1 groups, most genes were downregulated (54.8% and 50.4%, respectively) (Fig. 8).

**Fig. 9:**
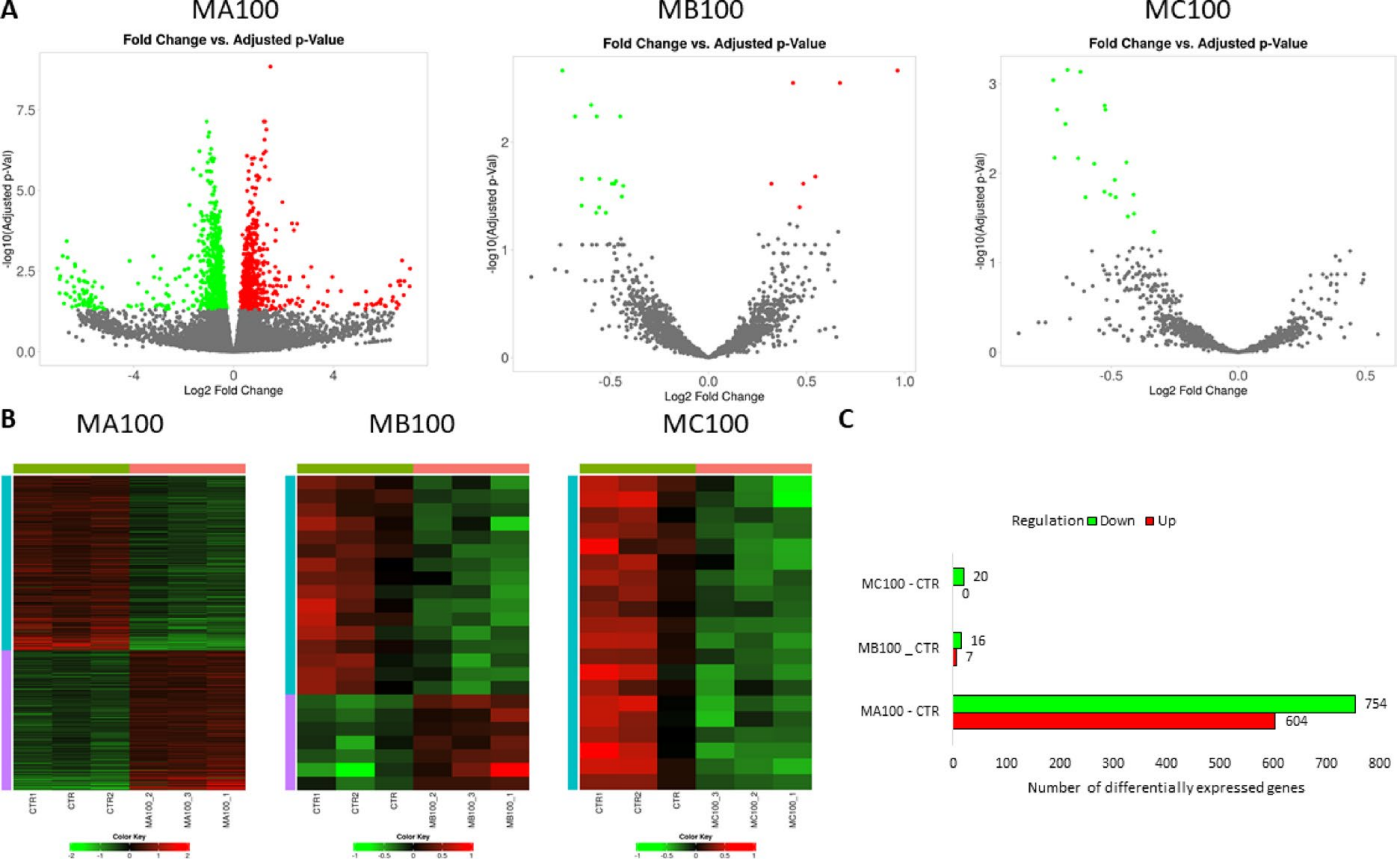
Results of differential expression analysis among HPF cells treated with different concentrations of 100 nm PS particles. (**A**) – Volcano plot for all analyzed genes (significantly altered genes (FDR < 0.05) are marked in red/green); (**B**) – Heatmaps for differentially expressed genes; (**C**) – Number of differentially expressed genes and their regulation.

Among the groups treated with 100 nm PS particles, the MA100 group had the highest number of DEGs (*n* = 1358) levels relative to CTR, and only a few genes with altered expression were found for the MB100 (*n* = 23) and MC100 (*n* = 20) groups (Supplementary File 2). For all three groups, the majority of genes were upregulated (55.5%, 69.6% and 100% respectively for MA100, MB100 and MC100) (Fig. 9).

**Fig. 10:**
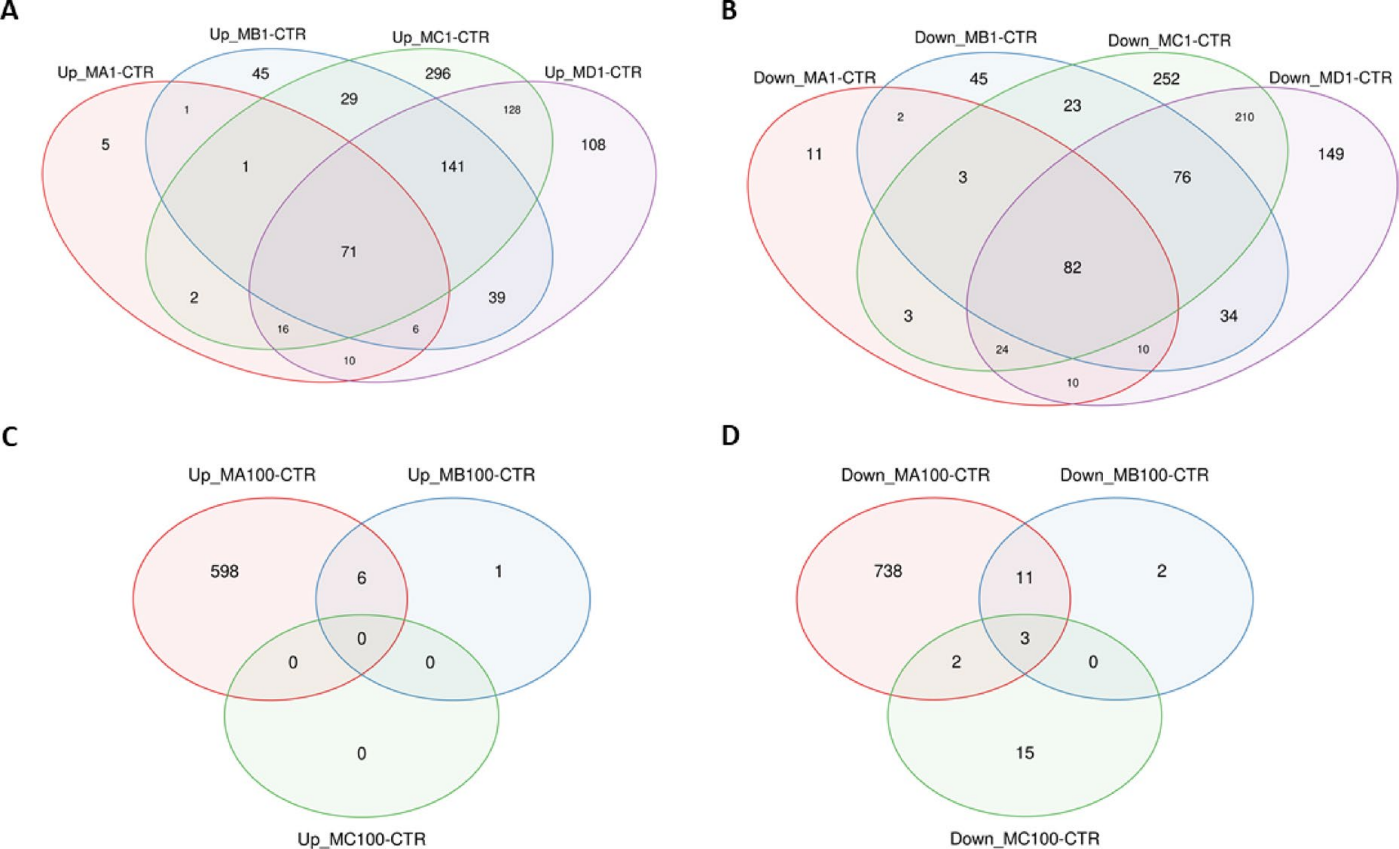
Venn diagrams for genes altered by different MNPs treatments (sizes and concentrations). (**A** and **B**) – up-regulated and down-regulated genes alerted by different concentrations of 1 μm PS particles, respectively; (**C** and **D**) – up-regulated and down-regulated genes alerted by different concentrations of 100 nm PS particles, respectively.

Of the 1832 different genes whose expression was altered by treatment with different doses of 1 μm PS particles (Supplementary File 3, Table S1), 153 were affected by all applied MNPs concentrations, of which 71 were upregulated and 82 were downregulated. For HPFs treated with 100 nm PS particles, of the 1376 altered genes (Supplementary File 3, Table S2), only three were common for all treatments, and all three were downregulated. Venn diagrams for up- and downregulated genes altered by different concentrations of 1 μm and 100 nm particles are presented in Fig. 10.

**Fig. 11:**
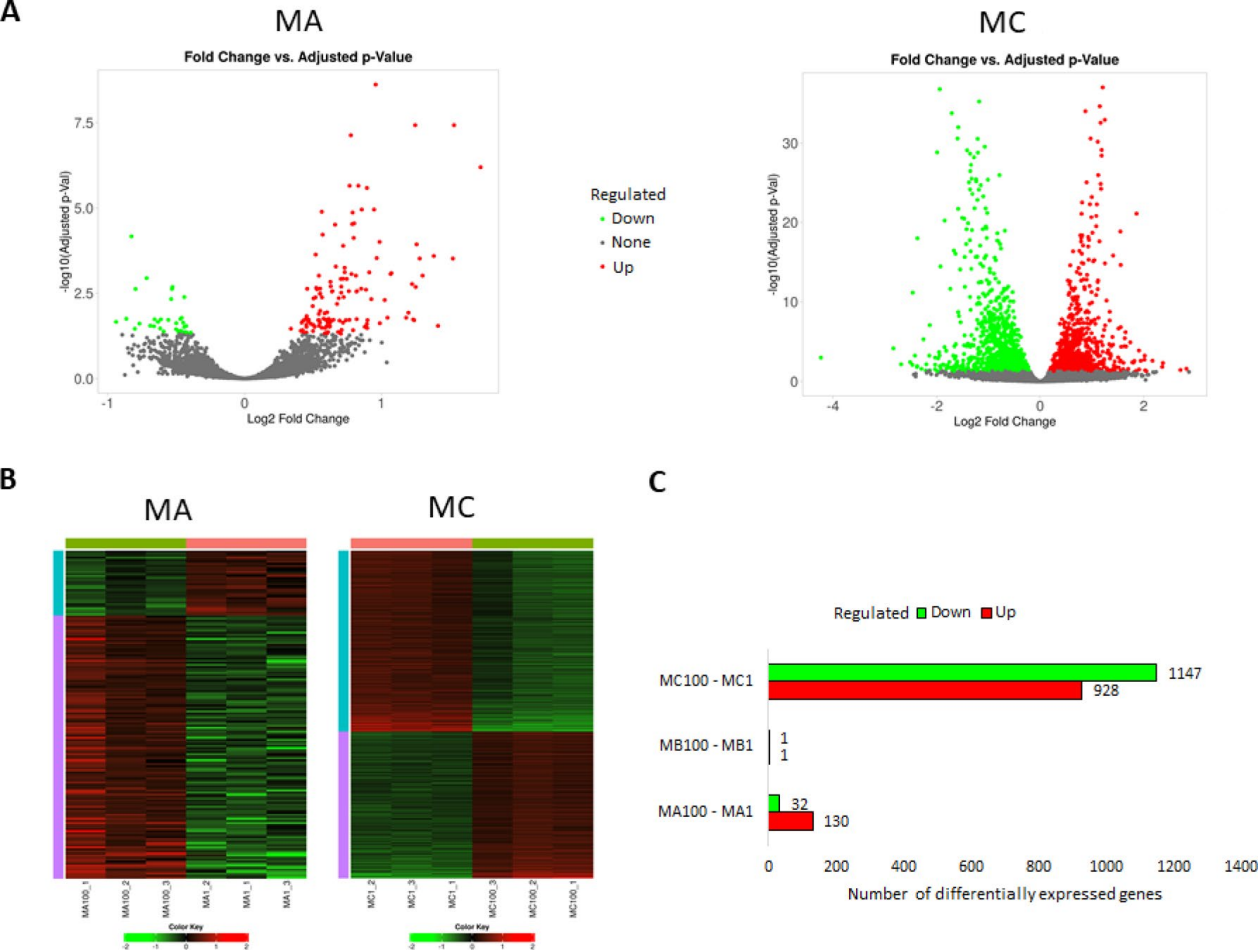
Results of differential expression analysis among HPF cells treated with the same concentrations of 1 μm and 100 nm microplastics particles. (**A**) – Volcano plot for all analyzed genes (significantly altered genes (FDR < 0.05) are marked in red/green); (**B**) – Heatmaps for differentially expressed genes; (**C**) – Number of differentially expressed genes and their regulation.

Differences in gene expression between cells treated with PS particles of the same concentration but differing in size were also assessed, using the groups exposed to 1 μm particles as a reference (Fig. 11). At the MA concentration, 162 DEGs were identified, of which 80.2% were upregulated and 19.8% downregulated. Only two DEGs were identified for the MB concentration group, with one gene being upregulated (hairy and enhancer of split-1, *HES1*) and the other downregulated (cathepsin, *CTSD*). In contrast, exposure to the MC concentration resulted in 2075 DEGs, with 44.7% upregulated and 55.3% downregulated (Supplementary File 4). Only one DEG was common to all applied concentrations and consistently upregulated. Among the 134 DEGs common to both MA and MC concentrations, two genes were consistently upregulated and one gene was downregulated. The remaining shared genes displayed opposite directions of regulation by MA and MC MNPs concentrations (Supplementary File 5).

### Functional annotation of genes altered by PS particles in HPFs

After exposure of HPF cells to different concentrations of 1 μm PS particles, a total of 1832 different genes were affected, ranging from 1357 (MC1) to 257 (MA1) per treatment. The overrepresentation tests for those genes in GO BP and KEEG categories are presented in Supplementary File 6.

Among the top 20 overrepresented (FDR < 0.05) BP for all applied concentrations of 1 μm particles, both up- and down-regulated processes were found. Comparative analysis of GO BP for all groups treated with different concentrations of 1 μm PS particles showed that among the processes overrepresented by up-regulated genes, regulation of catabolic process and regulation of cellular catabolic process were common. In turn, among the processes enriched by down-regulated genes observed in all groups treated with different concentrations of 1 μm MPs, there were processes such as cytoplasmic translation, translation, peptide biosynthesis and metabolism, organonitrogen compound biosynthesis, and biosynthesis and cellular metabolism of amides. Moreover, the up-regulated genes enriched processes such as macromolecule localization, negative regulation of protein metabolic process, autophagy, apoptotic process, protein localization, negative regulation of cellular metabolic process, cellular protein localization and positive regulation of metabolic process. The latter processes were overrepresented by genes altered by at least three different groups treated with 1 μm particles. In turn, down-regulated genes enriched processes such as oxidative phosphorylation, aerobic respiration, cellular respiration, ATP synthesis coupled electron transport, mitochondrial ATP synthesis coupled electron transport, ATP metabolic process, respiratory electron transport chain, aerobic electron transport chain, energy derivation by oxidation of organic compounds, electron transport chain, proton motive force-driven mitochondrial ATP synthesis and cellular macromolecule biosynthetic process. The processes were also overrepresented by genes altered in at least three different groups treated with 1 μm MPs.

For all applied concentrations of plastics with a particle size of 1 μm, the overrepresented (FDR < 0.05) KEGG pathways were enriched by down-regulated genes (from 11 pathways for the MB1 group to 18 for the MD1 group). The up-regulated genes enriched only apoptosis pathway for MD1 group. Among the pathways enriched by down-regulated genes for all groups treated with 1 μm MPs, there were: ribosome, coronavirus disease-COVID-19, Parkinson disease, prion disease, Huntington disease, oxidative phosphorylation, amyotrophic lateral sclerosis, Alzheimer disease, pathways of neurodegeneration-multiple diseases, proteasome. What is more, pathways such as: diabetic cardiomyopathy, protein export, thermogenesis, chemical carcinogenesis-reactive oxygen species, non-alcoholic fatty liver disease, cardiac muscle contraction were overrepresented by genes altered by at least three different concentrations.

For the experiment with 100 nm PS MPs, only genes altered in the MA100 groupoverrepresented (FDR < 0.05) BP pathways (both up and down regulated genes). Among the processes associated with up-regulated genes, the most significantly enriched were regulation of cell death, regulation of apoptotic process, regulation of programmed cell death, positive regulation of macromolecule metabolic process, and apoptotic process. In turn, among the down-regulated processes, cytoplasmic translation, oxidative phosphorylation, organonitrogen compound biosynthetic process, translation, and aerobic respiration were most significantly enriched. For the MB100 and MC100 groups, no significant enrichments of the GO Biological Processes database terms were detected.

Overrepresented (FDR < 0.05) KEGG pathways (for down-regulated genes) were detected only for MA100 (17 pathways) and MB100 (6 pathways) groups. The chemical carcinogenesis-reactive oxygen species pathway was common between these groups, for which in MB100 this enrichment was not statistically significant (FDR < 0.0777). For the group treated with MC100 MPs, no significant enrichment based on the KEGG database was detected. The overrepresentation tests for genes affected by exposure of HPF cells to different concentrations of 100 nm PS particles in GO BP and KEEG categories are presented in Supplementary File 7.

**Fig. 12:**
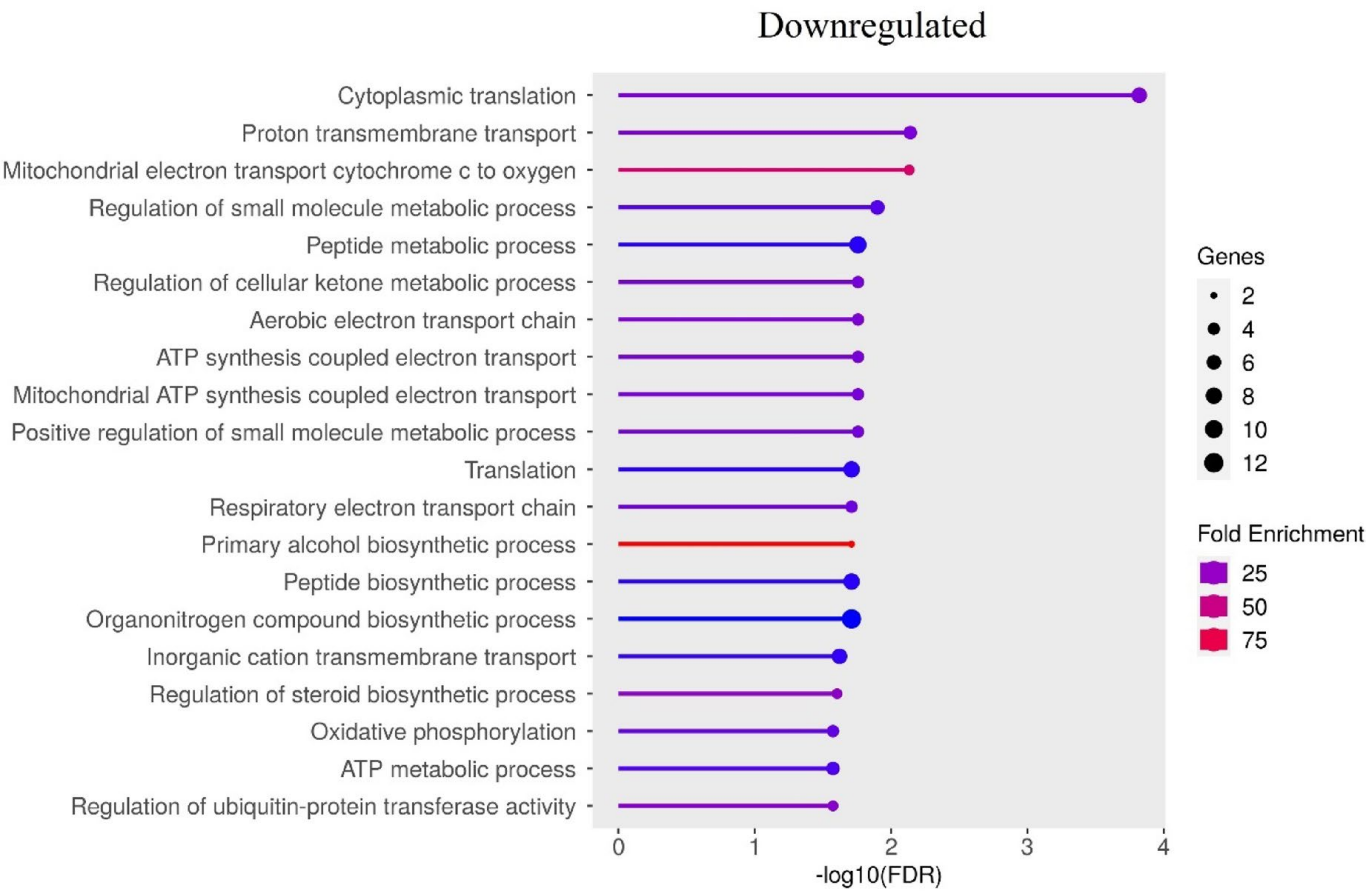
Top 20 biological processes enriched with genes altered by 100 nm microplastics particle treatment compared with the 1 μm microplastics particle treatment at the same MA concentrations (following remove redundant gene sets option). Only significantly enriched terms with FDR < 0.05 (Benjamini–Hochberg correction) are shown.

Comparison of different particle sizes at the highest applied concentrations (MA100 vs. MA1) revealed that significant GO Biological Process enrichment was limited to downregulated genes (Fig. 12, FDR < 0.05).

**Fig. 13:**
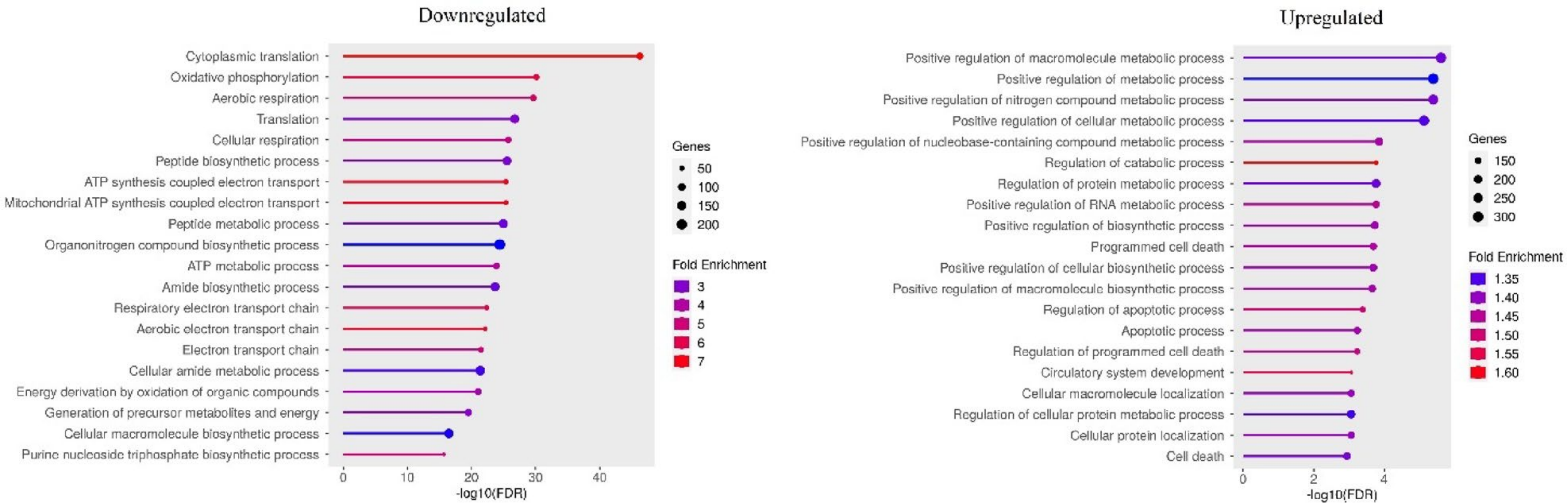
Top 20 biological processes enriched with genes altered by 100 nm microplastics particle treatment compared with the 1 μm microplastics particle treatment at the same MC concentrations (following remove redundant gene sets option). Only significantly enriched terms with FDR < 0.05 (Benjamini–Hochberg correction) are shown.

At the lowest concentrations (MC100 vs. MC1), significant enrichment was observed for both upregulated and downregulated GO Biological Process terms (Supplementary File 8). Notably, 11 out of the top 20 the most significantly overrepresented down-regulated BP in the MA comparison were found to be upregulated in the MC concentrations comparison. These shared pathways included: cytoplasmic translation, peptide metabolic process, aerobic electron transport chain, ATP synthesis coupled electron transport, mitochondrial ATP synthesis coupled electron transport, translation, respiratory electron transport chain, peptide biosynthetic process, organonitrogen compound biosynthetic process, oxidative phosphorylation, and ATP metabolic process. In the comparison of MC100 vs. MC1, among the top 20 overrepresented by down-regulated genes (Fig. 13, FDR < 0.05) GO BP, several processes were identified, including those associated with the positive regulation of metabolic processes, regulation of catabolic processes, positive regulation of biosynthetic processes, as well as pathways related to programmed cell death and the apoptotic process.

For MA MNPs concentration, the overrepresented (FDR < 0.05) KEGG pathways were associated only for down-regulated genes (3 pathways). For MC MNPs concentration, overrepresented (FDR < 0.05) KEGG pathways were also connected only with up-regulated genes (19 pathways). Ribosome, Parkinson disease and Coronavirus disease-COVID-19 pathways were common between both MA and MC concentrations, however, they were characterized by the opposite direction of regulation (Supplementary File 8).

### Validation of RNA-Seq expression by ddPCR

To assess the consistency between droplet digital PCR (ddPCR) quantification and RNA-seq expression levels, Pearson correlation coefficients were calculated for each gene. A moderate and statistically significant correlation was observed for *SOX9* (*r* = 0.65, *p* < 0.05) and *SOX4* (*r* = 0.63, *p* < 0.05), indicating good agreement between the two platforms. *FOXC1* also showed a moderate correlation (*r* = 0.49, *p* < 0.05), reflecting a similar expression pattern across methods despite slightly lower correlation strength. These results support the reliability of the RNA-seq data for all three genes, as validated by ddPCR.

## Discussion

Investigating the biological effects of MNPs in the pulmonary context requires the use of cellular models that reflect both structural and functional aspects of lung tissue. HPFs were selected in this study as a biologically relevant model to investigate cellular responses to MNPs exposure in the pulmonary environment. As resident stromal cells of the lung, HPFs are involved in tissue remodeling, oxidative stress regulation, and fibrotic processes, all of which are known to be affected by environmental pollutants^43^. These cells are known to respond to a variety of inhalable materials, including engineered nanoparticles such as TiO₂, ZnO, or silver, which can induce inflammatory and profibrotic effects even at subtoxic levels^44,45^. Since 100 nm particles fall within the nanoscale range of airborne pollutants and may penetrate into the alveolar interstitium, and 1 μm MPs have also been shown to deposit in small bronchi and lower airways under realistic breathing conditions^46^, HPFs offer a relevant model to investigate the molecular consequences of such exposures beyond the epithelial surface.

To characterize the full spectrum of size-dependent molecular responses, we focused the discussion on two concentrations that most clearly illustrated contrasting transcriptional profiles for each particle size. The lowest concentration tested in RNA-seq (0.001 g/L) represents the adaptive end of the response range and is discussed first. The highest concentration in the set (0.1 g/L) is associated with transcriptional suppression and is addressed later in the text. Intermediate concentrations followed the same overall trend, supporting the patterns described here and therefore not requiring separate discussion. At the low-exposure condition, we observed a distinct enrichment of energy-related pathways, particularly those associated with oxidative phosphorylation (GO:0006119) and ribosomal function (GO:0042254).

In cells exposed to 100 nm particles, we observed coordinated upregulation of mitochondrial and translational genes, suggesting an early metabolic shift. Notably, *NDUFS8* (encoding a core subunit of complex I in the electron transport chain, log2FC = 0.63, FDR = 3.43 × 10^–3^), *COX6A1* (cytochrome c oxidase subunit, log2FC = 0.61, FDR = 1.05 × 10^–3^), *ATP5F1B* (ATP synthase beta subunit, log2FC = 0.48, FDR = 3.39 × 10^–6^), *PRDX2* (a thioredoxin peroxidase involved in ROS detoxification, log2FC = 1.91, FDR = 1.30 × 10^–4^), and *RPL6* (a component of the large ribosomal subunit, log2FC = 0.57, FDR = 7.05 × 10^–3^) were significantly upregulated (Fig. 12). These genes are key players in electron transport and redox regulation, and their increased expression may indicate enhanced mitochondrial activity and a compensatory response to mild oxidative stress^47^. Likewise, the upregulation of *RPL6* and other ribosomal genes suggests stimulation of biosynthetic capacity, potentially linked to cellular adaptation rather than damage^48^. This pattern supports the hypothesis that smaller particles (100 nm), internalizing into HPFs can trigger a metabolic activation response aimed at maintaining homeostasis under environmental challenge. This interpretation is supported by Raman microscopy data and the results of performed cluster analysis, which confirmed the presence of PS inside HPFs.

Remarkably, raising the MNPs concentration to 0.1 g/L altered the previously observed transcriptional activation profile. While 100 nm NPs at lower doses triggered upregulation of mitochondrial and biosynthetic genes, the higher concentration led to a marked suppression of key metabolic and translational pathways, particularly in response to 1 μm MPs (MA-1). Among the most significantly downregulated pathways were cytoplasmic translation (GO:0002181) and oxidative phosphorylation (GO:0006119). Genes such as *RPS20* (40 S ribosomal protein S20; log2FC = − 0.54, FDR = 4.69 × 10^–2^), *RPS2* (40 S ribosomal protein S2; log2FC = − 0.56, FDR = 1.91 × 10^–2^), *RPS3A* (40 S ribosomal protein S3a; log2FC =–0.63, FDR = 2.19 × 10⁻²), and *RPL13* (60 S ribosomal protein L13a; log2FC = − 0.47, FDR = 1.80 × 10⁻²) — key components of the ribosomal machinery — showed reduced expression, indicating a potential suppression of translational activity. Similarly, downregulation of mitochondrial genes like *COX5A* (cytochrome c oxidase subunit 5 A; log2FC = − 0.48, FDR = 4.50 × 10⁻²), *COX5B* (subunit 5B of cytochrome c oxidase; log2FC = − 0.59, FDR = 4.17 × 10⁻²), and *MT-CO2* (mitochondrially encoded cytochrome c oxidase subunit II; log2FC =–0.39, FDR = 4.50 × 10⁻²) suggests impaired electron transport and ATP synthesis. Additional suppression of proton transmembrane transport (GO:1902600) further supports the notion of mitochondrial dysfunction, as genes such as *COX5B*, *MT-CO2*, and *PARK7* (DJ-1 protein, involved in redox regulation, log2FC =–0.45, FDR = 4.33 × 10⁻²) are critical for maintaining proton gradients and redox balance (Fig. 13). *PARK7*, in particular, has been shown to mitigate oxidative damage in lung epithelial and endothelial cells, acting as a protective factor during lung injury^49^.

Notably, we also observed reduced expression of *INSIG1* (insulin induced gene 1 protein, log2FC =–0.62, FDR = 3.38 × 10⁻²) and *EDEM1* (ER degradation enhancing alpha-mannosidase-like protein 1, log2FC =–0.94, FDR = 2.18 × 10⁻²) involved in the regulation of protein exit from the endoplasmic reticulum (GO:0070861), suggesting that high-dose exposure to 1 μm MPs may interfere with ER-to-Golgi trafficking and protein processing. EDEM1 is a well-established factor in ER-associated degradation (ERAD), promoting the clearance of misfolded proteins during ER stress^50^, and dysfunction in this system has been implicated in fibrotic progression within pulmonary fibroblasts^51^. In parallel, genes regulating ubiquitin-protein transferase activity (GO:0051438) such as *PARK7*, *RPS2*, and *RPS20* were also downregulated, indicating potential impairment of proteostasis. Together, these findings highlight how high-dose MPs exposure may compromise ER function and protein homeostasis in lung fibroblasts — a mechanism increasingly recognized in pulmonary fibrosis and stress responses^52^.

In summary, our data show that both particle size and concentration critically influence the cellular response to MNPs in human lung fibroblasts. At low concentrations (0.001 g/L), smaller particles (100 nm) induced transcriptional activation of mitochondrial and biosynthetic genes, suggesting a form of early metabolic adaptation. In contrast, at high concentrations (0.1 g/L), especially for larger particles (1 μm), we observed widespread suppression of metabolic, mitochondrial and proteostatic pathways, pointing to functional impairment and potential cellular stress. While our study is based on a single cell type, HPFs represent a widely accepted and physiologically relevant in vitro model for exploring tissue remodeling, oxidative stress responses, and fibrotic processes in the lung. Moreover, our transcriptomic findings were reinforced by complementary experimental approaches. Raman microscopy confirmed distinct patterns of particle interaction and/or internalization for 100 nm, 1 μm, and 5 μm MNPs. In parallel, phenotypic assays conducted at multiple time points (6, 24, and 48 h) provided additional temporal insight into the cellular responses, helping contextualize the observed gene expression changes. Although transcriptomic profiling alone does not capture downstream protein-level effects or functional endpoints, it offers a high-resolution snapshot of early cellular signaling and regulation. Taken together, the combined use of dose- and size-specific exposure, time-course functional assays, and Raman imaging strengthens the biological relevance of our findings. These considerations highlight typical limitations of in vitro transcriptomic studies, while also underscoring the value of integrating multi-angle approaches to enhance biological relevance.

The results obtained in the study clearly showed that Raman microscopy is an effective tool for tracking the process of MNPs internalization and the fate in cells. Despite the spatial resolution of slightly below a micrometer, together with cluster analysis, the method proved useful even for detecting 100 nm PS particles and/or their aggregates in HPFs. Studies on the internalization of MNPs into HPFs cells should be continued. In particular, it should be verified how this process depends on the dose and time of treatment to PS particles, as in this work the fate of MNPs was studied for the typically used 24-hour exposure and at the highest dose evaluated in toxicity and transcriptomic studies. Furthermore, it is necessary to optimize the protocol for preparing cells for Raman analysis, as well as the selection of equipment and measurement conditions, to enable a definitive determination of whether micrometer-sized particles are internalized into the cells.

While our study provides valuable insights into the cellular response to well-defined, laboratory-grade PS MNPs, several limitations must be considered when interpreting these findings in the context of environmentally relevant plastics. Raman microscopy, although highly specific and non-destructive, has reduced sensitivity for detecting highly weathered or biofouled particles. Moreover, the method may struggle with complex environmental matrices containing heterogeneous mixtures of polymers and inorganic materials. In terms of cytotoxicity and transcriptomic analyses, the use of pristine, spherical PS particles does not fully replicate the chemical and physical diversity of environmental MNPs, which can vary in shape, polymer composition, size distribution, and surface contaminants (e.g., heavy metals, organic pollutants, biofilms). These factors can significantly influence particle-cell interactions and cellular responses. Therefore, while our results offer a controlled model for understanding particle size-dependent effects, extrapolation to real-world exposure scenarios requires caution.

## Supplementary Information

Below is the link to the electronic supplementary material.

Supplementary Material 1

Supplementary Material 2

Supplementary Material 3

Supplementary Material 4

Supplementary Material 5

Supplementary Material 6

Supplementary Material 7

Supplementary Material 8

Supplementary Material 9

## Data Availability

The resulting raw sequences from the first sequencing read and read counts were deposited in the Gene Expression Omnibus (GEO) and Sequence Read Archive (SRA) databases of the National Center for Biotechnology Information (NCBI) under the accession number GSE296007. Supplementary materials, including additional figures, are provided in the online version of the article. All other datasets generated and/or analyzed during the current study are available from the corresponding author upon reasonable request.

## References

[1] Commission Regulation (EU). 2023/2055 of 25 September 2023 amending Annex XVII to regulation (EC) 1907/2006 of the European Parliament and of the Council concerning the Registration, Evaluation, authorisation and restriction of chemicals (REACH) as regards synthetic polymer microparticles (Text with EEA relevance). https://eur-lex.europa.eu/legal-content/EN/TXT/?uri=CELEX:32023R (2055).

[2] Song, J., Wang, C. & Li, G. Defining primary and secondary microplastics: A connotation analysis. *ACS ES&T Water*. **4** (6), 2330–2332 (2024).

[3] Ramsperger, A. F. R. M. et al. Nano- and microplastics: a comprehensive review on their exposure routes, translocation, and fate in humans. *NanoImpact* **29**, 100441 (2023). 36427812 10.1016/j.impact.2022.100441

[4] Leonard, V. L. et al. Microplastics in human blood: polymer types, concentrations and characterisation using µFTIR. *Environ. Int.* **188**, 108751 (2024). 38761430 10.1016/j.envint.2024.108751

[5] Kaushik, A., Singh, A., Kumar Gupta, V. & Mishra, Y. K. Nano/micro-plastic, an invisible threat getting into the brain. *Chemosphere* **361**, 142380 (2024). 38763401 10.1016/j.chemosphere.2024.142380

[6] Qin, X. et al. Features, potential invasion Pathways, and reproductive health risks of microplastics detected in human uterus. *Environ. Sci. Technol.* **58** (24), 10482–10493 (2024). 38829656 10.1021/acs.est.4c01541

[7] Paul, I., Mondal, P., Haldar, D. & Halder, G. Beyond the cradle – Amidst microplastics and the ongoing peril during pregnancy and neonatal stages: A holistic review. *J. Hazard. Mater.* **469**, 133963 (2024). 38461669 10.1016/j.jhazmat.2024.133963

[8] Wang, C., Hou, M., Shang, K., Wang, H. & Wang, J. Microplastics (Polystyrene) exposure induces metabolic changes in the liver of rare minnow (Gobiocypris rarus). *Molecules* **27** (3), 584 (2022). 35163849 10.3390/molecules27030584 PMC8840292

[9] Schwabl, P. et al. Detection of various microplastics in human stool. *Ann. Intern. Med.* **171** (7), 453–457 (2019). 31476765 10.7326/M19-0618

[10] Jenner, L. C. et al. Detection of microplastics in human lung tissue using µFTIR spectroscopy. *Sci. Total Environ.* **831**, 154907 (2022). 35364151 10.1016/j.scitotenv.2022.154907

[11] Baeza-Martínez, C. et al. First evidence of microplastics isolated in European citizens’ lower airway. *J. Hazard. Mater.* **438**, 129439 (2022). 35777146 10.1016/j.jhazmat.2022.129439

[12] Borgatta, M. & Breider, F. Inhalation of Microplastics—A toxicological complexity. *Toxics* **12** (5), 358 (2024). 38787137 10.3390/toxics12050358 PMC11125820

[13] Zhang, J. et al. Polystyrene microplastics induce pulmonary fibrosis by promoting alveolar epithelial cell ferroptosis through cGAS/STING signaling. *Ecotoxicol. Environ. Saf.* **277**, 116357 (2024). 38677073 10.1016/j.ecoenv.2024.116357

[14] Sun, X. et al. Mechanism of circRNA_SMG6 mediating lung macrophage ECM degradation via miR-570-3p in microplastics-induced emphysema. *Environ. Int.* **187**, 108701 (2024). 38685156 10.1016/j.envint.2024.108701

[15] Feng, Y. et al. A systematic review of the impacts of exposure to micro- and nano-plastics on human tissue accumulation and health. *Eco-Environment Health*. **2** (4), 195–207 (2023). 38435355 10.1016/j.eehl.2023.08.002 PMC10902512

[16] Jin, W. et al. Microplastics exposure causes the senescence of human lung epithelial cells and mouse lungs by inducing ROS signaling. *Environ. Int.* **185**, 108489 (2024). 38367553 10.1016/j.envint.2024.108489

[17] Goodman, K. E., Hare, J. T., Khamis, Z. I., Hua, T. & Sang, Q. X. A. Exposure of human lung cells to polystyrene microplastics significantly retards cell proliferation and triggers morphological changes. *Chem. Res. Toxicol.* **34** (4), 1069–1081 (2021). 33720697 10.1021/acs.chemrestox.0c00486

[18] Yadav, S. et al. Recent analytical techniques, and potential eco-toxicological impacts of textile fibrous microplastics (FMPs) and associated contaminates: A review. *Chemosphere* **326**, 138495 (2023). 36963588 10.1016/j.chemosphere.2023.138495

[19] Huang, Z., Hu, B. & Wang, H. Analytical methods for microplastics in the environment: a review. *Environ. Chem. Lett.* **21** (1), 383–401 (2023). 36196263 10.1007/s10311-022-01525-7 PMC9521859

[20] Liza, A. A. et al. Microplastic pollution: a review of techniques to identify microplastics and their threats to the aquatic ecosystem. *Environ. Monit. Assess.* **196** (3), 285 (2024). 38374279 10.1007/s10661-024-12441-4

[21] Karlsson, T. M., Kärrman, A., Rotander, A. & Hassellöv, M. Comparison between Manta trawl and in situ pump filtration methods, and guidance for visual identification of microplastics in surface waters. *Environ. Sci. Pollut. Res.* **27** (5), 5559–5571 (2020). 10.1007/s11356-019-07274-5 PMC7028838 31853844

[22] Luo, X., Wang, Z., Yang, L., Gao, T. & Zhang, Y. A review of analytical methods and models used in atmospheric microplastic research. *Sci. Total Environ.* **828**, 154487 (2022). 35278538 10.1016/j.scitotenv.2022.154487

[23] Lavers, J. L., Oppel, S. & Bond, A. L. Factors influencing the detection of beach plastic debris. *Mar. Environ. Res.* **119**, 245–251 (2016). 27363010 10.1016/j.marenvres.2016.06.009

[24] Dekiff, J. H., Remy, D., Klasmeier, J. & Fries, E. Occurrence and Spatial distribution of microplastics in sediments from Norderney. *Environ. Pollut.* **186**, 248–256 (2014). 24448461 10.1016/j.envpol.2013.11.019

[25] Taudul, B., Tielens, F. & Calatayud, M. Raman characterization of plastics: A DFT study of polystyrene. *J. Phys. Chem. B*. **128** (17), 4243–4254 (2024). 38632700 10.1021/acs.jpcb.3c08453

[26] Zhi, Z., Li, Y., Liu, G. & Ou, Q. Identification and detection of label-free polystyrene microplastics in maize seedlings by Raman spectroscopy. *Sci. Total Environ.* **958**, 178093 (2025). 39693673 10.1016/j.scitotenv.2024.178093

[27] Liu, Y. et al. Overcoming the fluorescent interference during Raman spectroscopy detection of microplastics. *Sci. Total Environ.* **897**, 165333 (2023). 37414187 10.1016/j.scitotenv.2023.165333

[28] ApoTox-Glo™ Triplex Assay Protocol. https://www.promega.com/resources/protocols/technical-manuals/101/apotox-glo-triplex-assay-protocol/. (Accessed 06.05.2025).

[29] Męczyńska-Wielgosz, S., Sikorska, K., Czerwińska, M., Kapka-Skrzypczak, L. & Kruszewski, M. Uptake and toxicity of polystyrene NPs in three human cell lines. *Int. J. Mol. Sci.* **26** (10), 4783 (2025). 40429925 10.3390/ijms26104783 PMC12111990

[30] Poma, A. et al. In vitro genotoxicity of polystyrene nanoparticles on the human fibroblast Hs27 cell line. *Nanomaterials* **9** (9), 1299 (2019). 31514347 10.3390/nano9091299 PMC6781270

[31] El Hayek, E. et al. Photoaging of polystyrene microspheres causes oxidative alterations to surface physicochemistry and enhances airway epithelial toxicity. *Toxicol. Sci.* **193** (1), 90–102 (2023). 36881996 10.1093/toxsci/kfad023 PMC10176241

[32] Park, K. Y., Kim, M. S. & Oh, N. Cytotoxicity of amine-modified polystyrene MPs and NPs on neural stem cells cultured from mouse subventricular zone. *Heliyon* **10** (10), e30518 (2024). 38770330 10.1016/j.heliyon.2024.e30518 PMC11103427

[33] Dodt, M., Roehr, J., Ahmed, R. & Dieterich, C. FLEXBAR—Flexible barcode and adapter processing for Next-Generation sequencing platforms. *Biology (Basel)*. **1** (3), 895–905 (2012). 24832523 10.3390/biology1030895 PMC4009805

[34] Dobin, A. et al. STAR: ultrafast universal RNA-seq aligner. *Bioinformatics* **29** (1), 15–21 (2013). 23104886 10.1093/bioinformatics/bts635 PMC3530905

[35] Anders, S., Pyl, P. T. & Huber, W. HTSeq—a python framework to work with high-throughput sequencing data. *Bioinformatics* **31** (2), 166–169 (2015). 25260700 10.1093/bioinformatics/btu638 PMC4287950

[36] Love, M. I., Huber, W. & Anders, S. Moderated Estimation of fold change and dispersion for RNA-seq data with DESeq2. *Genome Biol.* **15** (12), 550 (2014). 25516281 10.1186/s13059-014-0550-8 PMC4302049

[37] Kanehisa, M., Furumichi, M., Sato, Y., Matsuura, Y. & Ishiguro-Watanabe, M. KEGG: biological systems database as a model of the real world. *Nucleic Acids Res.* **53** (D1), D672–D677 (2025). 39417505 10.1093/nar/gkae909 PMC11701520

[38] Ge, S. X., Son, E. W. & Yao, R. iDEP: an integrated web application for differential expression and pathway analysis of RNA-Seq data. *BMC Bioinform.* **19** (1), 534 (2018). 10.1186/s12859-018-2486-6 PMC6299935 30567491

[39] Xu G, Cheng H, Jones R, Feng Y, Gong K, Li K, et al. Surface-Enhanced Raman Spectroscopy Facilitates the Detection of Microplastics <1 µm in the Environment. *Environ. Sci. Technol.* (2020). 10.1021/acs.est.0c02317 33095569

[40] Tian, M. et al. Direct identification and visualisation of real-world contaminating microplastics using Raman spectral mapping with multivariate curve resolution-alternating least squares. *J. Hazard. Mater.* **422**, 126892 (2022). 34425427 10.1016/j.jhazmat.2021.126892

[41] Noda, L. K. & Sala, O. A resonance Raman investigation on the interaction of styrene and 4-methyl styrene oligomers on sulphated titanium oxide. *Spectrochim Acta Mol. Biomol. Spectrosc.* **56** (1), 145–155 (2000). 10.1016/s1386-1425(99)00128-6 10728865

[42] Dong, M. et al. Raman spectra and surface changes of microplastics weathered under natural environments. *Sci. Total Environ.* **739**, 139990 (2020). 32535468 10.1016/j.scitotenv.2020.139990

[43] Zhang, H., Li, M., Hu, C. J. & Stenmark, K. R. Fibroblasts in pulmonary hypertension: roles and molecular mechanisms. *Cells* **13** (11), 914 (2024). 38891046 10.3390/cells13110914 PMC11171669

[44] Lee, M. K. H. et al. 3D airway Epithelial–Fibroblast biomimetic microfluidic platform to unravel engineered Nanoparticle-Induced acute stress responses as exposome determinants. *Environ. Sci. Technol.* **57** (48), 19223–19235 (2023). 37933439 10.1021/acs.est.3c03678

[45] Löfdahl, A. et al. Silver nanoparticles alter cell viability ex vivo and in vitro and induce Proinflammatory effects in human lung fibroblasts. *Nanomaterials* **10** (9), 1868 (2020). 32961914 10.3390/nano10091868 PMC7557856

[46] Huang, X., Saha, S. C., Saha, G., Francis, I. & Luo, Z. Transport and deposition of microplastics and nanoplastics in the human respiratory tract. *Environ. Adv.* **16**, 100525 (2024).

[47] Gu, W. et al. Single-Cell RNA sequencing reveals Size-Dependent effects of polystyrene microplastics on immune and secretory cell populations from zebrafish intestines. *Environ. Sci. Technol.* **54** (6), 3417–3427 (2020). 32092251 10.1021/acs.est.9b06386

[48] Saenen, N. D. et al. Polystyrene microplastics of varying sizes and shapes induce distinct redox and mitochondrial stress responses in a Caco-2 monolayer. *Antioxidants* **12** (3), 739 (2023). 36978987 10.3390/antiox12030739 PMC10045319

[49] Amatullah, H. et al. Protective function of DJ-1/PARK7 in lipopolysaccharide and ventilator-induced acute lung injury. *Redox Biol.* **38**, 101796 (2021). 33246293 10.1016/j.redox.2020.101796 PMC7695876

[50] Katsuki, R., Kanuka, M., Ohta, R., Yoshida, S. & Tamura, T. Turnover of < scp > EDEM1, an < scp > ERAD -enhancing factor, is mediated by multiple degradation routes. *Genes Cells*. **29** (6), 486–502 (2024). 38682256 10.1111/gtc.13117 PMC11163939

[51] Baek, H. A. et al. Involvement of Endoplasmic reticulum stress in myofibroblastic differentiation of lung fibroblasts. *Am. J. Respir Cell. Mol. Biol.* **46** (6), 731–739 (2012). 21852685 10.1165/rcmb.2011-0121OC

[52] Lenna, S. & Trojanowska, M. The role of Endoplasmic reticulum stress and the unfolded protein response in fibrosis. *Curr. Opin. Rheumatol.* **24** (6), 663–668 (2012). 22918530 10.1097/BOR.0b013e3283588dbb PMC3828639

