# Supplementary material for: Tracing micro and nanoplastics toxicity in human pulmonary fibroblasts through integrated Raman and transcriptomic analyses
Source: Sci Rep. 2025 Nov 11;15:39459. doi: 10.1038/s41598-025-22947-7 (PMC12606128; doi:10.1038/s41598-025-22947-7)
Supplement: Supplementary file 9 — Supplementary Material 9 [file 41598_2025_22947_MOESM9_ESM.docx]

**Supplementary File 1.** Sequencing reads and mapping statistics.

**Supplementary File 2.** Differential expression analysis for HLF cells treated with different concentrations of 1 µm and 100 nm microplastic particles (including gene regulation tables).

**Supplementary File 3.** Comparative analysis for genes affected by different microplastic particle concentrations (including altered gene annotations). Table S1. Genes affected by different concentrations of 1 µm microplastic particles. Table S2. Genes affected by different concentrations of 100 nm microplastic particles.

**Supplementary File 4.** Differential expression analysis for HLF cells treated with the same concentrations of 1 µm and 100 nm microplastic particles (including gene regulation annotations).

**Supplementary File 5.** Comparative analysis for genes affected by the same concentrations of 1 µm and 100 nm microplastic particles (including altered gene annotations).

**Supplementary File 6.** Altered genes (by all 1 µm microplastic particle concentrations separately) overrepresentation tests in GO biological processes and KEGG pathways categories (Top 100 categories). Pathway annotations are based on the KEGG database (www.kegg.jp)

**Supplementary File 7.** Altered genes (by all 100 nm microplastic particle concentrations separately) overrepresentation tests in GO biological processes and KEGG pathways categories (Top 100 categories). Pathway annotations are based on the KEGG database (www.kegg.jp)

**Supplementary File 8.** Altered genes (by all 100 nm microplastic particle concentrations separately) overrepresentation tests in GO biological processes and KEGG pathways categories (Top 100 categories). Pathway annotations are based on the KEGG database (www.kegg.jp)
